# Reconciling Experiment and Theory in the Use of Aryl-Extended Calix[4]pyrrole Receptors for the Experimental Quantification of Chloride–π Interactions in Solution

**DOI:** 10.3390/ijms16048934

**Published:** 2015-04-22

**Authors:** Antonio Bauzá, David Quiñonero, Antonio Frontera, Pablo Ballester

**Affiliations:** 1 Departament de Química, Universitat de les Illes Balears, 07122 Palma de Mallorca, Spain; E-Mails: antonio.bauza@uib.es (A.B.); david.quinonero@uib.es (D.Q.); 2 Catalan Institution for Research and Advanced Studies (ICREA), 08018 Barcelona, Spain; 3 Institute of Chemical Research of Catalonia (ICIQ), 43007 Tarragona, Spain

**Keywords:** anion–π interaction, DFT calculations, binding energies

## Abstract

In this manuscript we consider from a theoretical point of view the recently reported experimental quantification of anion–π interactions (the attractive force between electron deficient aromatic rings and anions) in solution using aryl extended calix[4]pyrrole receptors as model systems. Experimentally, two series of calix[4]pyrrole receptors functionalized, respectively, with two and four aryl rings at the *meso* positions, were used to assess the strength of chloride–π interactions in acetonitrile solution. As a result of these studies the contribution of each individual chloride–π interaction was quantified to be very small (<1 kcal/mol). This result is in contrast with the values derived from most theoretical calculations. Herein we report a theoretical study using high-level density functional theory (DFT) calculations that provides a plausible explanation for the observed disagreement between theory and experiment. The study reveals the existence of molecular interactions between solvent molecules and the aromatic walls of the receptors that strongly modulate the chloride–π interaction. In addition, the obtained theoretical results also suggest that the chloride-calix[4]pyrrole complex used as reference to dissect experimentally the contribution of the chloride–π interactions to the total binding energy for both the two and four-wall aryl-extended calix[4]pyrrole model systems is probably not ideal.

## 1. Introduction

Non-covalent interactions are prominent players in supramolecular chemistry and biochemistry dominating the many processes of living systems and dictating the functionality of many biological and host-guest systems [1,2,3,4,5,6]. The complete understanding of the different non-covalent forces is essential for the rational design of new drugs and developing improved synthetic receptors capable to function in competitive media. Interactions involving π-systems in general and aromatic rings in particular are very relevant in supramolecular chemistry [7,8,9,10]. A very well-known example is the cation–π interaction [11] that is important in determining the structure of protein and participates in enzyme catalysis [12,13]. Traditionally, the π-system is considered as an electron rich (π-basic) binding block. The naissance of the counterintuitive anion–π interaction [14,15,16] that can be defined as the attractive interaction between an anion and an electron poor π-system (π-acid) was somewhat controversial [17,18]. However, in the last decade a great deal of theoretical and experimental investigations has time-honored the anion–π interaction as an important supramolecular bond. Its nature has been studied by a many theoretical studies [19,20,21,22] in addition to an increasing amount of experimental investigations [23,24,25,26,27]. Anion–π interactions have become noticeable players in fields as diverse as medicine, environmental chemistry and biochemical processes [14,28,29,30]. Moreover, their application to the design of highly selective anion receptors, transport channels [31] and catalysis [32,33] definitively confirms their significance in the field of supramolecular chemistry [34,35].

The design and synthesis of selective receptors for anion binding is a topic of continuous interest [36,37,38,39,40,41]. The main reason is the vital function played by anions, which are ubiquitous throughout biological systems [42,43]. In addition, some anions are increasingly recognized as problematic environmental contaminants [44,45]. Interestingly, a new class of anion receptors based on the anion–π interaction is emerging in the literature [14,46]. For instance, an interesting receptor, that combines hydrogen bonding and anion–π interaction for the binding of anions with neutral π-acceptors, has been recently published by Albrecht and collaborators [47] and its ability to trap anions has been demonstrated both in solution and in the solid state.

**Figure 1:**
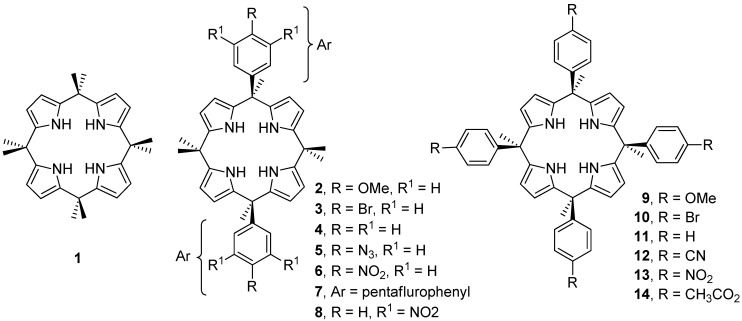
Molecular structures of receptors **1–14** studied in this work.

Recently, some of us used neutral receptors in an attempt to assess the energy of anion–π interactions [48,49,50]. Two series of *meso*-tetraaryl and *meso*-diaryl calix[4]pyrrole receptors were used as model systems to quantify chloride–π interactions in solution. The chloride–arene interactions observed in these complexes are established exclusively with the π–aromatic system as demonstrated by means of ^1^H-NMR spectroscopy and X-ray crystallography. The plots of either sigma Hammett (σ_p_) values or electrostatic surface potential (ESP) values at the center of the phenyl rings *vs.* measured free-energies showed nice linear relationships and were used to demonstrate that the detected chloride–π interactions must be dominated by electrostatic effects. Unexpectedly, the values of the attractive free energies quantified for the interaction of chloride with π–aromatic systems having ESPs values at its center larger than zero were very modest (about 1.1 kcal/mol per aromatic ring). The quantification of the chloride–π interactions was performed by using the free-energy value of the chloride complex with octamethyl calix[4]pyrrole (Cl^−^@**1**, see Figure 1) as an ideal reference. The free energy for the formation of the latter complex was attributed to the hydrogen bonding interactions established between the chloride anion and the four NHs of the calix[4]pyrrole core. In addition, the strength of this primary interaction was supposed to be maintained constant throughout the receptors series. In short, the difference in binding energy between “two wall” (**2–10**, see Figure 1) or “four-wall” (**11–17**) Cl^−^ complexes and the “no-wall” reference Cl^−^@**1** (ΔΔG values) represented the contribution to the overall binding exerted by the interaction between the “two/four walls” of receptors and the chloride anion. Theoretical studies demonstrated that anion–π interactions were cumulative, thus the value for a single chloride–π interaction was experimentally approximated by simply dividing the measured magnitude differences from the model systems by the number of aromatic rings involved, two or four (statistical correction).

In this manuscript we report a theoretical DFT study where we analyze the unexpectedly modest anion–π interaction values measured experimentally in solution. This study reveals that solvent effects may play a very important role in the observed discrepancy between theory and experiment. We also examine the influence of the presence of additional interactions to hydrogen–bonding in the complex of octamethylcalix[4]pyrrole with chloride Cl^−^@**1** used as reference. The consideration of additional interactions in the reference complex should affect the values of the experimental estimates for the chloride–π interaction. We computed the interaction energies of the chloride complexes with receptors **1–14** (see Figure 1) and compared them to the values measured experimentally. In addition, we analyzed by means of Bader’s theory of “atoms in molecules” and noncovalent interaction (NCI) plots the presence of important C–H···Cl^−^ interactions in the Cl^−^@**1** complex used as reference for the two model systems. The existence of such interactions helps to explain the modest energy values determined experimentally for chloride–π interaction using the two aryl-extended calix[4]pyrrole model systems.

## 2. Results and Discussion

### 2.1. Solvation Effects

The experimental ΔΔG values of the Cl^−^ complexes are summarized in Table 1 for both two-wall and four-wall receptors (see Figure 1). In addition we have also included the ΔΔE values computed at the BP86-D3/def2-TZVP level of theory for comparison purposes. The ΔE values are computed using the following equation:

CH_3_CN@receptor + Cl^−^ → Cl^−^@receptor + CH_3_CN (1)

That is, since the X-ray structures of the free four-wall aryl-extended calix[4]pyrrole receptors incorporate an acetonitrile molecule forming four CH_3_CN···HN_pyrrole_ hydrogen bonds, we have evaluated energetically the process of replacing this acetonitrile molecule by a chloride for these receptors and we have used the same procedure for the two aryl-extended calix[4]pyrrole ones mainly for comparison purposes. The ΔΔE values summarized in Table 1 are calculated subtracting to the ΔE value computed for the chloride reference complex using Equation (1) the ΔE values corresponding to complexes **2–14** measured using Equation (1) and dividing the results by 4 or 2 depending on the number of aromatic walls present in the complex. The mentioned statistical correction of the ΔΔE and ΔΔG values is not fully justifiable thermodynamically but simplifies the data analysis and comparison. Thus by comparing the statistically corrected experimental ΔΔG_exp_(SC) and theoretical ΔΔE_theor_(SC) values, it can be observed that the experimentally determined magnitudes are significantly smaller. In fact, all statistically corrected ΔΔG_exp_(SC) values are ≤1 kcal/mol. These values are experimentally significant based on the differences measured for the association constants of the complexes, however, they are within the theoretical accuracy error and consequently very difficult to reproduce theoretically. The theoretically calculated values are larger in absolute value than the experimental ones. As a matter of fact theory seems to overestimate attraction by a factor close to 10 and repulsion between 2- and 3-fold with respect to the experimental magnitudes. The magnitudes of the experimental values, ΔΔG_exp_(SC), determined for the repulsive chloride–π interactions using the four-wall model systems are slightly larger than those obtained employing the two-wall analogue. Most likely, the repulsive interactions cannot be avoided or minimized in the four-wall receptors owing to conformational restraints of the cone conformation. That is, in the four wall receptors the separation of two distal *meso*-phenyl groups away from the chloride to avoid repulsion induces the closeness of the two adjacent ones. In contrast, for the two wall receptors a related conformational change brings the two *meso*-methyl groups in close proximity to the chloride.

**Figure 2:**
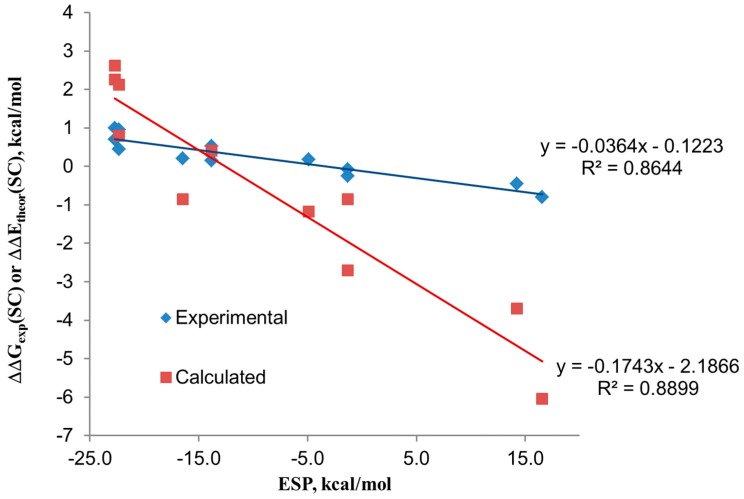
Regression plots of the statistically corrected (SC) experimental ΔΔG and theoretical ΔΔE magnitudes *vs.* the electrostatic surface potential (ESP) value at the center of the aryl rings. The ESP values were calculated at the RI-MP2/aug-cc-pVDZ level of theory for a single substituted aryl ring.

The linear relationships observed for the two *pseudo*-Hammet plots (experimental and theoretical statistically corrected free energies for two- and four-wall receptors *vs.* the electrostatic potential values measured over the center of the arene rings) indicate that both theory and experiment are in support of the described chloride–π interactions being mainly driven by electrostatics. However, the magnitude of the electrostatic term is clearly overestimated by the calculation compared to experiment as indicated by the magnitudes of the slopes of the corresponding plots (see Figure 2).

**Figure 3:**
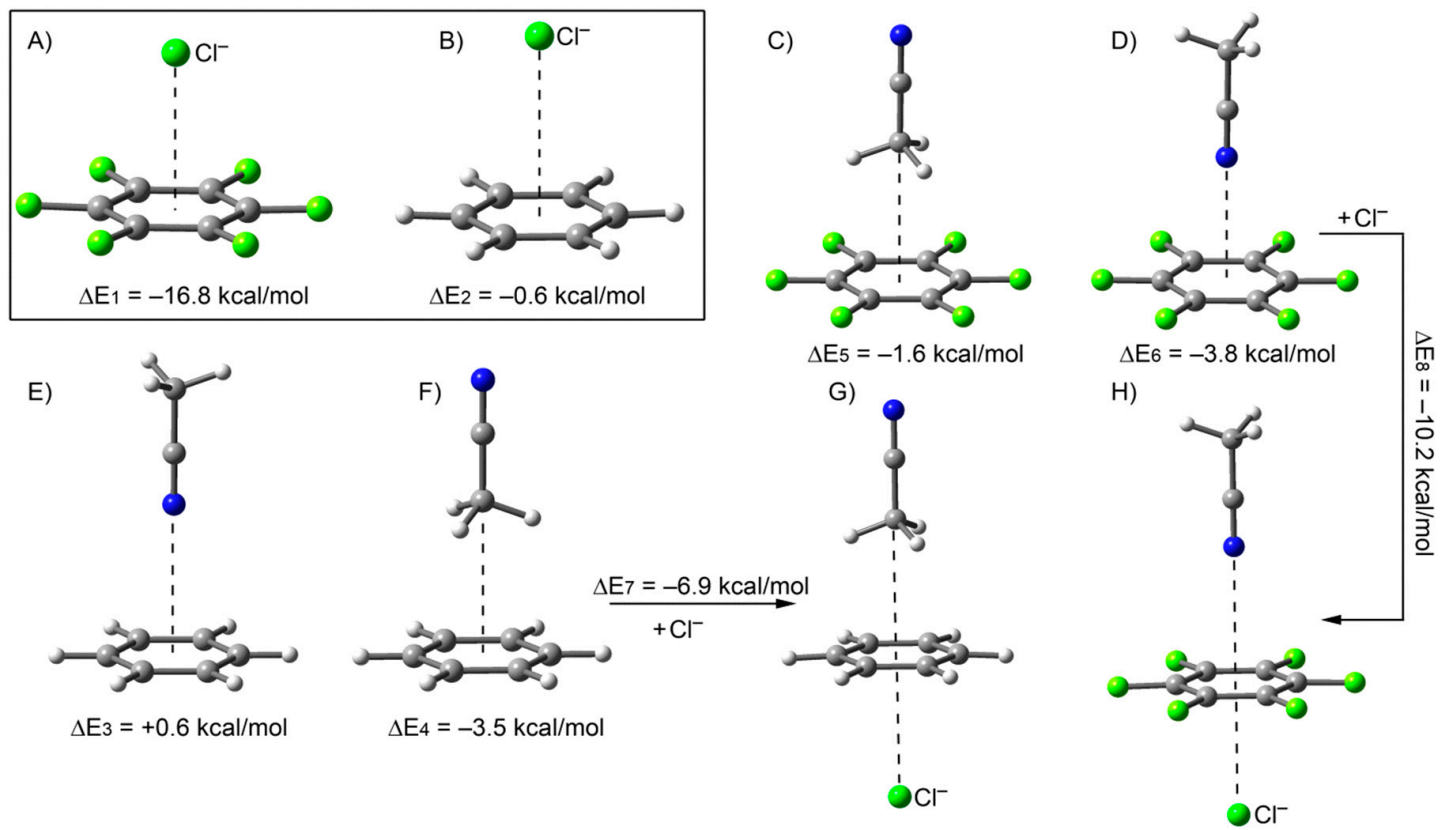
Energy optimized geometries of some anion–π, lp–π, C–H···π model complexes and the corresponding interaction energy values (kcal/mol). (**A**) Hexafluorobenze·chloride complex stabilized by anion–π interaction; (**B**) Benzene·chloride complex stabilized by anion–π interaction; (**C**) Hexafluorobenzene·acetonitrile complex stabilized by C–H···π interactions; (**D**) Hexafluorobenzene·acetonitrile complex stabilized by lp–π interactions; (**E**) Benzene·acetonitrile complex stabilized by lp–π interactions; (**F**) Benzene·acetonitrile complex stabilized by C–H···π interactions; (**G**) Termolecular complex of benzene with acetonitrile and chloride stabilized by CH–π and anion–π interactions, respectively; (**H**) Termolecular complex of hexafluororobenzene with acetonitrile and chloride stabilized by lp–π and anion–π interactions, respectively.

In general, as mentioned above, there is not good agreement between theory and experiment in the magnitudes of the binding energies, however, in most cases (10 out of 12) the calculated sign of the interaction (negative is attractive, positive is repulsive) coincided. The disagreement is probably due to solvation effects in the binding process that are not considered in the calculations (the experimental data were measured in CH_3_CN). In addition, the anion–π interaction strength is influenced by the type of interaction that the aromatic ring establishes in the opposite face. Some of us previously demonstrated that the formation of a CH···π interaction enhanced the ability of the π-system to participate in an anion–π interaction at the opposite face [51,52,53]. The contrary occurs when the aromatic ring participates in lp–π interactions prior to anion binding. We performed a preliminary theoretical study where we compared the influence of both interactions (C–H···π and lp–π) on the chloride–π interaction using acetonitrile as C–H/lp donor molecule and benzene/*hexa*-fluorobenzene as π-systems. The results are summarized in Figure 3 and some very interesting conclusions can be drawn from our results. Firstly, the electron rich aromatic ring (represented by benzene) forms energetically more favorable C–H···π than lp–π complexes with acetonitrile (see Figure 3E,F). Therefore the outer surface of receptors with electron rich aromatic walls (electron donating substituents) are preferentially solvated by establishing C–H···π interactions with acetonitrile molecules. Conversely, the electron deficient phenyl rings (exemplified by *hexa*-fluorobenzene) form energetically more favorable lp–π than C–H···π interactions with acetonitrile molecules (see Figure 3C,D). Therefore the outer surface of the *meso*-aromatic walls of the receptors decorated with electron withdrawing substituents are likely solvated by means of lp–π interactions with solvent molecules. Secondly, the anion–π interaction energy of *hexa*-fluorobenzene with chloride (ΔE_1_ = −16.8 kcal/mol) is significantly reduced in absolute value when the acetonitrile is interacting at the opposite side of the π-system through the N atom (ΔE_8_ = −10.2 kcal/mol). This is because the nitrogen atom is donating electron density to the ring thus weakening the anion–π interaction. Third, the almost negligible anion–π interaction energy of benzene with chloride (ΔE_2_ = −0.6 kcal/mol) is significantly increased in absolute value if the acetonitrile is interacting at the opposite side of the π-system through the CH–π interactions (ΔE_8_ = −6.9 kcal/mol). The main reason is that the CH_3_ group withdraws electron density from the aromatic ring and thus strengthens the anion–π interaction at the opposite face. Taken together, these results indicate that the nature/type of the initial interaction of the aromatic ring with an acetonitrile molecule (solvent effects) dramatically modulated the binding energy associated to the subsequent anion–π interaction for both electron-rich and electron-poor aromatic rings. These results hint at the important effects of the solvent and solvation effects in explaining the small binding-energy values measured experimentally for the Cl^−^–π interactions using the two different model systems of *meso*-aryl receptors.

**Table 1:** Experimental ΔG, ΔΔG, and theoretical ΔΔE values in kcal/mol for the Cl^−^ complexes of the calix[4]pyrrole receptor series. See text for details. The statistically corrected ΔΔG(SC) and ΔΔE(SC) values are obtained by dividing ΔΔG and ΔΔE by the number of receptor walls.

Compound	Aryl	Walls	−ΔG_exp_	ΔΔG_exp_	ΔΔE_theor_	ΔΔG_exp_ (SC)	ΔΔE_theor_ (SC)
Cl^−^@**1**	none	none	6.9	–	–	–	–
Cl^−^@**2**	R = OMe, R^1^ = H	two	5.5	1.4	4.5	0.7	2.3
Cl^−^@**3**	R = Br, R^1^ = H	two	6.6	0.3	0.8	0.1	0.4
Cl^−^@**4**	R = R^1^ = H	two	6.0	0.9	1.6	0.4	0.8
Cl^−^@**5**	R = N_3_	two	6.5	0.4	−1.7	0.2	−0.8
Cl^−^@**6**	R = NO_2_	two	7.4	−0.5	−5.4	−0.2	−2.7
Cl^−^@**7**	C_6_F_5_	two	7.8	−0.9	−7.4	−0.4	−3.7
Cl^−^@**8**	R = H, R^1^ = NO_2_	two	8.5	−1.6	−12.1	−0.8	−6.0
Cl^−^@**9**	R = OMe	four	2.9	4.0	10.5	1.0	2.6
Cl^−^@**10**	R = Br	four	4.8	2.1	–	0.5	–
Cl^−^@**11**	R = H	four	3.1	3.8	8.5	0.9	2.1
Cl^−^@**12**	R = CN	four	6.2	0.7	−4.7	0.2	−1.2
Cl^−^@**13**	R = NO_2_	four	7.2	−0.3	−3.4	−0.1	−0.8
Cl^−^@**14**	R = CH_3_CO_2_	four	4.1	2.8	13.0	0.7	3.2

### 2.2. The Chloride Complex Cl^−^@**1** Used as Reference for Both Model Systems

When model systems are used to quantify weak intermolecular interactions one of the key issues is the selection of an adequate reference. Further examination of the results summarized in Table 1 revealed that the contribution of each chloride–π interaction to the overall binding energy is indeed very small. For instance, in the two-wall receptor series, receptor **8** provides the largest binding free energy for chloride and the contribution to it of the anion–π interaction was estimated as ΔΔG_exp_/2 = −0.8 kcal/mol per aromatic dinitro-phenyl ring. For the series of four walls receptors, the estimate of the chloride–π interaction using receptor **13** (the only one that improves the binding of the reference receptor) was ΔΔG_exp_/4 ~ −0.1 kcal/mol per nitro-phenyl ring. These unexpectedly small values estimated for the chloride–π interaction strength could be due to the existence of additional secondary favorable interactions in the chloride complex Cl*^−^*@**1** selected as reference to subtract the hydrogen bonding contribution. If this was the case, the magnitude of the favorable secondary interactions must be taken into account and subtracted from the total binding energy of the chloride Cl*^−^*@**1** complex used as reference of the hydrogen bonding binding energy. An experimental result that provided some support to this hypothesis can be extracted from the data in Table 1. The estimated ΔΔG values for the chloride–π interactions that were operative in the complexes of receptors **6** (two-walls) and **13** (four-walls) were very similar (−0.5 and −0.3 kcal/mol, respectively). Taking into account that the aromatic wall substituent is the same in both complexes, R = NO_2_, but the number of aromatic walls is two for **6** and four for **13**, the almost equivalent free energy of binding measured for the chloride–π interactions in the two complexes could be explained considering that the two axial methyl groups of the *meso*-carbon atoms of **6** also interacted with the chloride and that the strength of this interaction is similar to that of the chloride–π interaction. In short, the four aromatic chloride–π interactions in the Cl*^−^*@**13** complex are equivalent to two aromatic chloride–π interactions plus two methyl(CH)···Cl^−^ interactions in the Cl*^−^*@**6** complex and also similar to the four methyl(CH)···Cl^−^ interactions in the reference Cl*^−^*@**1** complex.

We had originally considered an alternative explanation, which consisted of the idea that the interaction energies measured in acetonitrile solution for the methyl(CH)···Cl^−^ and the chloride–π interaction with the nitro-phenyl group were comparable and close to zero. Only in this latter case, the selection of the complex of chloride with octamethylcalix[4]pyrrole, Cl^*−*^@**1**, as reference can be considered as an ideal choice.

**Figure 4:**
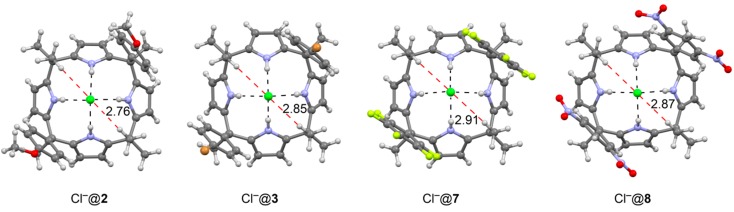
X-ray geometries of chloride complexes with receptors **2**, **3**, **7** and **8**. Distances are indicated in Å. The CH···Cl^−^ interactions are marked using a red dashed line.

The X-ray structure of the octamethylcalix[4]pyrrole **1** bound to Cl^−^ is not available; however several chloride complexes with two-wall receptors have been characterized by X-ray crystallography. The structures are included in Figure 4 and the experimental methyl(C–H)···Cl^−^ distances range from 2.76 to 2.91 Å (measured from H to Cl). At these distances, charge-dipole interactions are certainly present and, consequently, a favorable contribution for these interactions to the overall binding energy must be anticipated.

**Figure 5:**
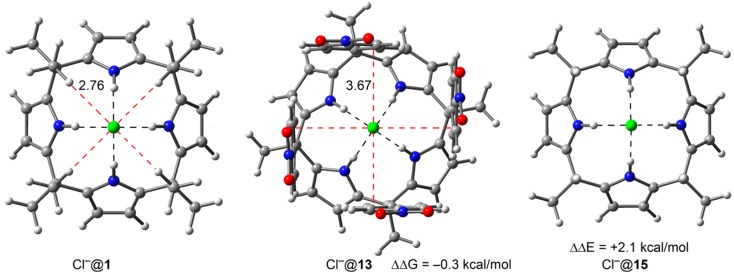
DFT energy optimized chloride complexes with receptors **1**, **13** and **15**. Distances in Å. The CH···Cl^−^ and anion–π interactions are indicated using red dashed lines.

The optimized geometry of the complexes exhibited C–H···Cl^−^ distances that are comparable to the X-ray ones, ranging from 2.71 to 2.86 Å. Figure 4 depicts the optimized geometry complex Cl^−^@**1** and the averaged C–H···Cl^−^ distance in it is 2.76 Å. In Figure 5 we also show the optimized geometry of complex Cl^−^@**13** (four wall receptor with R = NO_2_). It can be observed that the anion–π distance in Cl^−^@**13** is longer (~3.7 Å) than the calculated optimum for a Cl^−^···π distance (~3.2 Å). Probably, this represents another contributing factor to the small ΔΔG values measured experimentally using the two calix[4]pyrrole model systems. Clearly, the location of the Cl anion in the complex is mainly determined by the four strong hydrogen bonds that are established with the four pyrrole NH groups. The limited conformational flexibility exhibited by the calix[4]pyrrole core does not allow a significant modification of this long distance and consequently the anion–π interactions in the model systems are expected to be weaker than theoretically predicted. In addition, the reference complex might provide four weak C–H···Cl^−^ hydrogen bonds that are not present in the Cl^−^@**13** complex. Therefore, the experimentally estimated ΔΔG value of −0.3 kcal/mol could simply mean that the four anion–π interactions are 0.3 kcal/mol more favorable than the four C–H···Cl^−^ hydrogen bonds. Thus, this magnitude did not correspond to a direct estimate of the chloride–π interaction, which should be somewhat larger.

We propose here the use of another chloride complex as “*in silico*” reference (receptor **15**, see Figure 5, right). In the new reference, four *meso*-methyl groups are replaced with hydrogen atoms. Unfortunately, this receptor is not easily accessible synthetically. In addition, the conformation of the chloride complex of receptor **15**, having the four methyl groups axially oriented, is energetically more favorable. Nevertheless, we computed the ΔΔE value of the Cl^−^@**15** complex with respect to that of the chloride complex with the reference receptor **1**, Cl*^−^*@**1**, and the result was 2.1 kcal/mol in favor of the latter. Therefore, a rough estimation of the anion–π interactions contributing to the overall energy of complex Cl^−^@**13** would be on the order of −2.4 kcal/mol (*i.e.*, −0.3 + (−2.1)) assuming that the lack of the four C–H···Cl^−^ interactions of the reference are equivalent to the 2.1 kcal/mol calculated above. Using this new reference receptor **15**, each individual chloride–π interaction present in the Cl^−^@**13** complex could be quantified to contribute to its total free energy with—0.6 kcal/mol. This new estimated value for the chloride–π interaction energy of a *p*-nitro-phenyl group is still very modest even compared to the weak C–H···π interaction but closer to the theoretically predicted value. As mentioned above, the small estimates of the chloride–π interactions that emerged from the two aryl-extended calix[4]pyrrole model systems can be related to the long anion–π distance observed in the corresponding complexes and to strong solvation effects occurring in solution. As a matter of fact, a recent investigation [54] determined the gas-phase association energy of the anion–π interactions between tetraoxacalix[2]arene[2]triazine **1** with Cl^−^ (among other anions) using a time-of-flight (TOF) mass spectrometer equipped with an electrospray ionization source, a cryogenically controlled 3D Paul trap and a magnetic-bottle TOF photoelectron analyzer. The measured experimental interaction energy for each chloride–π contact using this methodology was ~16 kcal/mol.

**Figure 6:**
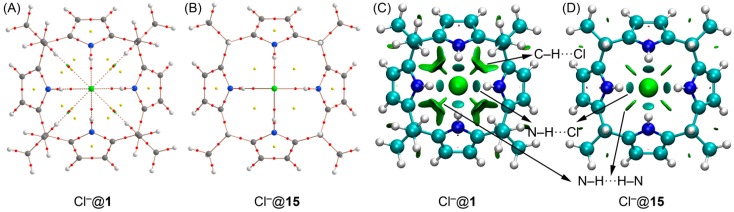
The “atoms-in-molecules” (AIM) analyses of chloride complexes with receptors **1** and **15** (**A**,**B**, respectively). Bond, ring and cage critical points are represented by red, yellow and green spheres, respectively. NCI plots of complexes Cl^−^@**1** and Cl^−^@**15** (**C**,**D**, respectively). Distances are indicated in Å.

In order to confirm that C–H···Cl^−^ hydrogen bonds existed in the Cl^−^@**1** complex used as reference we performed a combined “atoms-in-molecules” and noncovalent interaction (NCI) plot study of complexes Cl^−^@**1** and Cl^−^@**15** (see Figure 6). The distribution of critical points in the complex Cl^−^@**1** showed four bond critical points and bond paths symmetrically distributed that connected the chloride with the four N–H groups. In addition, it showed four bond critical points and bond paths that connected the chloride anion with four C–H bonds. The value of the charge density was higher at the bond critical points that characterize the N–H···Cl hydrogen bond than that at the bond critical points of the C–H···Cl bonds, in good agreement with their relative strength. In our theoretically proposed reference chloride complex (receptor **15**), only four symmetrically distributed critical points were generated upon complexation of Cl^−^ that corresponds to the N–H···Cl hydrogen bonds. We also used the NCI plot to study the C–H···Cl^−^ interactions observed in the Cl^−^@**1** complex experimentally used as reference. The NCI plot is a visualization index based on the electron density and its derivatives, and enables identification and visualization of non-covalent interactions efficiently. The isosurfaces correspond to both favorable and unfavorable interactions, as differentiated by the sign of the second density Hessian eigenvalue are defined by the isosurface color. NCI analysis allows an assessment of host–guest complementarity and the extent to which weak interactions contribute to stabilize the complex. The information provided by NCI plots is essentially qualitative, *i.e.*, which molecular regions interact. The color scheme is a red–yellow–green–blue scale with red for ρ^+^_cut_ (repulsive) and blue for ρ^−^_cut_ (attractive). Yellow and green surfaces correspond to weak repulsive and weak attractive interactions, respectively. In Figure 6B,C we show the representation of the NCI plot computed for the complexes Cl^−^@**1** and Cl^−^@**15**. For both structures, several non-covalent regions clearly appear between the chloride and the receptor. For instance, four round blue isosurfaces were found between the anion and the N–H groups in both structures, which are characteristic of strong H–bonding interactions. Four small green isosurfaces also appeared between the N–H groups of the pyrroles in both complexes indicative of hydrophobic interactions. The main difference between the NCI plots of both complexes (Cl^−^@**1** and Cl^−^@**15**) is the presence of four symmetrically distributed green isosurfaces between the C–H and Cl^−^ groups in Cl^−^@**1** that clearly demonstrates the existence of attractive and weak C–H···Cl^−^ hydrogen bonding interactions.

## 3. Experimental Section

The energies of the complexes included in this study were optimized at the BP86-D3/def2-TZVP level of theory using the program TURBOMOLE version 6.4. [55] The interaction energies were calculated with correction for the basis set superposition error (BSSE) by using the Boys-Bernardi counterpoise technique [56]. For the calculations we have used the BP86 functional with the latest available correction for dispersion (D3). The “atoms-in-molecules” (AIM) [57] analysis was performed at the BP86/def2-TZVP level of theory. The calculation of AIM properties was made using the AIMAll program [58]. The cartesian coordinates of all the energy minimized structures discussed in the text are included in the supplementary materials.

We have used the NCI method [59,60,61] to study the CH–chloride interactions observed in the structures of the complexes Cl^−^@**1** and Cl^−^@**15**. This method relies on two scalar fields to map local bonding properties: the electron density (ρ) and the reduced-density gradient (RDG, s). It is able to map real-space regions where non-covalent interactions are important, and is based exclusively on the electron density and its gradient. The information provided by NCI plots is essentially qualitative, *i.e.*, which molecular regions interact. The color scheme is a red–green–blue scale with red for ρ^+^_cut_ (repulsive) and blue for ρ^−^_cut_ (attractive).

## 4. Conclusions

In this manuscript, we reanalyzed the available experimental data concerning the complexation of chloride by *meso*-phenyl substituted calix [4] pyrrole receptors providing new interpretations to the small free energies derived from them for chloride–π interactions. The explanations we provide here are based on the results of high level DFT studies. The solvation of one face of am aryl group by a molecule of acetonitrile strongly influences the subsequent chloride–π interaction occurring at its opposite face. The chloride–π interaction is weakened in electron deficient rings but strengthened in electron rich counterparts. Most likely, this solvation process occurs during the binding of chloride with the receptors, altering significantly the calculated energy differences for the corresponding complexes in gas-phase or without considering such explicit solvation. Secondly, the selection of the chloride complex used as reference for the experimental work is probably not ideal. Theoretical calculations suggest that in the chloride complex used as reference there are additional attractive interactions C–H···Cl^−^ aside of strong N–H···Cl hydrogen bonds. This finding was evidenced by means of AIM analyses and NCI plots. We propose adding the difference in energy computed for the chloride complexes of receptors **15** and **1**, ΔΔE_(Cl_−_@**15**–Cl_−_@**1**)_= −2.1 kcal/mol, to the experimentally determined values for the chloride–π interactions using the calix[4]pyrrole model systems in order to produce more realistic estimates. In receptor **15**, four axial *meso*-methyl groups are replaced with hydrogen atoms compared to **1**. An additional explanation for the very week energy contribution of chloride–π interactions emerging from the use of aryl-extended calix[4]pyrrole model systems has to do with the long chloride–π distance featured in the corresponding chloride complexes. Finally, the experimentally estimated values for the chloride–π interaction energies can be re-interpreted as the energy difference between pure chloride–π interactions and theoretically not trivial C–H···Cl^−^ interactions. If this was the case, the attractive energies assigned experimentally to single chloride–π interactions ought to be increased by *ca.* 0.5 kcal/mol.

## Supplementary Materials

Supplementary materials can be found at http://www.mdpi.com/1422-0067/16/04/8934/s1.
