# Supplementary material for: Reconciling Experiment and Theory in the Use of Aryl-Extended Calix[4]pyrrole Receptors for the Experimental Quantification of Chloride–π Interactions in Solution
Source: Int J Mol Sci. 2015 Apr 22;16(4):8934–48. doi: 10.3390/ijms16048934 (PMC4425116; doi:10.3390/ijms16048934)
Supplement: Supplementary file 1 [file ijms-16-08934-s001.pdf]

Supplementary Information

Cl^{-}@**1**

N	−1.6861702	−1.6861702	0.2256400
N	−1.6861702	1.6861702	0.2256400
N	1.6861702	1.6861702	0.2256400
N	1.6861702	−1.6861702	0.2256400
C	0.0000000	−3.5865718	0.1054932
C	−1.2577147	−2.8648953	−0.3498651
C	−2.1905353	−3.1979546	−1.3227313
C	−3.1979546	−2.1905353	−1.3227313
C	−2.8648953	−1.2577147	−0.3498651
C	−3.5865718	0.0000000	0.1054932
C	−2.8648953	1.2577147	−0.3498651
C	−3.1979546	2.1905353	−1.3227313
C	−2.1905353	3.1979546	−1.3227313
C	−1.2577147	2.8648953	−0.3498651
C	0.0000000	3.5865718	0.1054932
C	1.2577147	2.8648953	−0.3498651
C	2.1905353	3.1979546	−1.3227313
C	3.1979546	2.1905353	−1.3227313
C	2.8648953	1.2577147	−0.3498651
C	3.5865718	0.0000000	0.1054932
C	2.8648953	−1.2577147	−0.3498651
C	3.1979546	−2.1905353	−1.3227313
C	2.1905353	−3.1979546	−1.3227313
C	1.2577147	−2.8648953	−0.3498651
H	−2.1568638	−4.0735670	−1.9629172
H	−4.0735670	−2.1568638	−1.9629172
H	−4.0735670	2.1568638	−1.9629172
H	−2.1568638	4.0735670	−1.9629172
H	2.1568638	4.0735670	−1.9629172
H	4.0735670	2.1568638	−1.9629172
H	4.0735670	−2.1568638	−1.9629172
H	2.1568638	−4.0735670	−1.9629172
C	5.0047771	0.0000000	−0.4939721
H	5.5515266	0.8944631	−0.1641724
H	5.5515266	−0.8944631	−0.1641724
H	4.9791512	0.0000000	−1.5921341
C	0.0000000	−5.0047771	−0.4939721
H	0.8944631	−5.5515266	−0.1641724
H	−0.8944631	−5.5515266	−0.1641724
H	0.0000000	−4.9791512	−1.5921341

C	−5.0047771	0.0000000	−0.4939721
H	−5.5515266	−0.8944631	−0.1641724
H	−5.5515266	0.8944631	−0.1641724
H	−4.9791512	0.0000000	−1.5921341
C	0.0000000	5.0047771	−0.4939721
H	−0.8944631	5.5515266	−0.1641724
H	0.8944631	5.5515266	−0.1641724
H	0.0000000	4.9791512	−1.5921341
H	−1.1878019	−1.1878019	0.9757038
H	1.1878019	−1.1878019	0.9757038
H	1.1878019	1.1878019	0.9757038
H	−1.1878019	1.1878019	0.9757038
Cl	0.0000000	0.0000000	2.4659909
C	0.0000000	3.7213293	1.6509510
H	−0.8961438	4.2702530	1.9768994
H	0.0000000	2.7463979	2.1573940
H	0.8961438	4.2702530	1.9768994
C	−3.7213293	0.0000000	1.6509510
H	−4.2702530	−0.8961438	1.9768994
H	−2.7463979	0.0000000	2.1573940
H	−4.2702530	0.8961438	1.9768994
C	3.7213293	0.0000000	1.6509510
H	2.7463979	0.0000000	2.1573940
H	4.2702530	−0.8961438	1.9768994
H	4.2702530	0.8961438	1.9768994
C	0.0000000	−3.7213293	1.6509510
H	−0.8961438	−4.2702530	1.9768994
H	0.8961438	−4.2702530	1.9768994
H	0.0000000	−2.7463979	2.1573940

CH_3CN@1

N	−2.6298491	−0.0147882	0.0599568
H	−1.7628979	−0.0266081	−0.4779343
C	−3.2830949	−1.1400340	0.5102245
C	−4.3937290	−0.7080308	1.2185607
H	−5.1214156	−1.3445014	1.7111947
C	−4.3900799	0.7207849	1.1975297
H	−5.1141764	1.3750743	1.6717322
C	−3.2773645	1.1264945	0.4769192
C	−2.6813708	2.4878348	0.1886104
N	−0.1371003	2.5515628	−0.0394315

H	−0.1536130	2.5565867	−1.0510574
C	−1.2670697	2.5058439	0.7494972
C	−0.8288823	2.4186546	2.0621582
H	−1.4715582	2.3434710	2.9331127
C	0.5933630	2.4205455	2.0500052
H	1.2442787	2.3778936	2.9171790
C	1.0128005	2.5074268	0.7284049
C	2.4028922	2.5430875	0.1283720
N	2.2194068	0.0022736	0.1829176
H	1.3154922	0.0000702	0.6545552
C	2.9261339	1.1386836	−0.1359901
C	4.1272124	0.7155675	−0.6977679
H	4.9237456	1.3632102	−1.0525091
C	4.1226229	−0.7094769	−0.7091759
H	4.9130276	−1.3559026	−1.0791731
C	2.9189711	−1.1335393	−0.1562657
C	2.3831484	−2.5391318	0.0764938
N	−0.1601444	−2.5757046	−0.0294374
H	−0.2002281	−2.6039087	−1.0400553
C	1.0070094	−2.5029116	0.7083326
C	0.6190724	−2.3854406	2.0374813
H	1.2908471	−2.3146297	2.8869062
C	−0.8024751	−2.3929098	2.0839875
H	−1.4245903	−2.2991406	2.9679578
C	−1.2712561	−2.5167243	0.7846677
C	−2.6970528	−2.5121607	0.2538638
C	−3.5171406	3.5832724	0.8796604
H	−4.5513177	3.5705211	0.5065543
H	−3.0814565	4.5708741	0.6768984
H	−3.5399026	3.4355809	1.9668037
C	3.3625585	3.2532423	1.1131809
H	3.4226850	2.7060590	2.0624595
H	3.0163109	4.2758427	1.3191491
H	4.3749129	3.2961810	0.6883819
C	3.3536481	−3.2930246	1.0175685
H	4.3575155	−3.3341321	0.5725751
H	2.9982523	−4.3177071	1.1955699
H	3.4379549	−2.7796012	1.9837289
C	−3.5218574	−3.5824993	0.9951711
H	−3.0928051	−4.5774668	0.8154619
H	−4.5630315	−3.5785342	0.6418488
H	−3.5234230	−3.4001033	2.0772825

C	0.6572128	−0.0032972	−2.6320127
N	−0.3503871	−0.0390817	−2.0544228
C	1.9175492	0.0474325	−3.3452035
H	1.9197868	0.8926047	−4.0468348
H	2.7399230	0.1713855	−2.6204625
H	2.0710976	−0.8839425	−3.9064287
C	2.3397236	−3.2843999	−1.2796460
H	3.3491690	−3.3450361	−1.7074470
H	1.9504241	−4.3049208	−1.1537148
H	1.7124599	−2.7543165	−2.0119756
C	−2.7336378	−2.8203056	−1.2617080
H	−2.2954036	−3.8074430	−1.4772016
H	−3.7761787	−2.8253564	−1.6057581
H	−2.2030482	−2.0562852	−1.8490993
C	−2.6868147	2.7502309	−1.3357881
H	−3.7222790	2.7470184	−1.7007123
H	−2.2425060	3.7300229	−1.5714906
H	−2.1462719	1.9688035	−1.8899930
C	2.3983028	3.3310606	−1.2043694
H	2.0160159	4.3507177	−1.0537089
H	3.4175225	3.3945486	−1.6083004
H	1.7823599	2.8325324	−1.9678396

Cl^{-}@**2**

Cl	0.0000000	0.0000000	−1.1543808
N	−1.7244281	−1.5924011	1.0676764
H	−1.2211994	−1.1285238	0.2939573
C	−1.3583125	−2.8081380	1.6027490
C	−2.3079965	−3.1321376	2.5614343
H	−2.3167124	−4.0292720	3.1720202
C	−3.2678971	−2.0785996	2.5902662
H	−4.1428554	−2.0235779	3.2297623
C	−2.8885434	−1.1336217	1.6449895
C	−3.5687209	0.1476239	1.1945942
N	−1.5802182	1.7217540	1.0791013
H	−1.0962281	1.1926457	0.3350874
C	−2.8011655	1.3860972	1.6234683
C	−3.1408401	2.4022412	2.5078213
H	−4.0418931	2.4466853	3.1110114
C	−2.0983480	3.3738955	2.4739089
H	−2.0583360	4.2957184	3.0450451

C	−1.1400703	2.9308245	1.5731221
C	0.1490826	3.5743456	1.0940974
N	1.7244281	1.5924011	1.0676764
H	1.2211994	1.1285238	0.2939573
C	1.3583125	2.8081380	1.6027490
C	2.3079965	3.1321376	2.5614343
H	2.3167124	4.0292720	3.1720202
C	3.2678971	2.0785996	2.5902662
H	4.1428554	2.0235779	3.2297623
C	2.8885434	1.1336217	1.6449895
C	3.5687209	−0.1476239	1.1945942
N	1.5802182	−1.7217540	1.0791013
H	1.0962281	−1.1926457	0.3350874
C	2.8011655	−1.3860972	1.6234683
C	3.1408401	−2.4022412	2.5078213
H	4.0418931	−2.4466853	3.1110114
C	2.0983480	−3.3738955	2.4739089
H	2.0583360	−4.2957184	3.0450451
C	1.1400703	−2.9308245	1.5731221
C	−0.1490826	−3.5743456	1.0940974
C	−4.9796321	0.2010851	1.8071674
H	−5.5588487	−0.6779476	1.4922803
H	−5.5013493	1.1080762	1.4711706
H	−4.9421026	0.2133560	2.9051377
C	0.2126769	5.0223930	1.6305108
H	0.1962995	5.0384570	2.7295027
H	−0.6476178	5.5932726	1.2573249
H	1.1363330	5.5049434	1.2857074
C	4.9796321	−0.2010851	1.8071674
H	5.5588487	0.6779476	1.4922803
H	5.5013493	−1.1080762	1.4711706
H	4.9421026	−0.2133560	2.9051377
C	−0.2126769	−5.0223930	1.6305108
H	0.6476178	−5.5932726	1.2573249
H	−1.1363330	−5.5049434	1.2857074
H	−0.1962995	−5.0384570	2.7295027
C	0.1840912	3.6542959	−0.4482819
C	1.4089110	3.7372704	−1.1304886
H	2.3386181	3.7072810	−0.5593363
C	1.4610839	3.8167753	−2.5169678
C	0.2759643	3.8134171	−3.2650117
C	−0.9555105	3.7614993	−2.6039018

C	−0.9875066	3.6875376	−1.2074116
H	−1.9528104	3.6242436	−0.7032758
C	−0.1840912	−3.6542959	−0.4482819
C	0.9875066	−3.6875376	−1.2074116
H	1.9528104	−3.6242436	−0.7032758
C	0.9555105	−3.7614993	−2.6039018
C	−0.2759643	−3.8134171	−3.2650117
C	−1.4610839	−3.8167753	−2.5169678
C	−1.4089110	−3.7372704	−1.1304886
H	−2.3386181	−3.7072810	−0.5593363
C	−3.7147380	0.1416139	−0.3516761
H	−4.2938540	−0.7387203	−0.6686446
H	−4.2381996	1.0527221	−0.6785675
H	−2.7427000	0.1091765	−0.8629016
C	3.7147380	−0.1416139	−0.3516761
H	4.2938540	0.7387203	−0.6686446
H	4.2381996	−1.0527221	−0.6785675
H	2.7427000	−0.1091765	−0.8629016
H	2.4144680	3.8523319	−3.0457118
H	−1.8938029	3.7504912	−3.1564834
H	−2.4144680	−3.8523319	−3.0457118
H	1.8938029	−3.7504912	−3.1564834
O	0.4290827	3.8582793	−4.6345335
O	−0.4290827	−3.8582793	−4.6345335
C	−0.7456614	3.7164151	−5.4236317
H	−0.4093918	3.7147242	−6.4678687
H	−1.2681488	2.7688590	−5.2057737
H	−1.4474896	4.5559331	−5.2712559
C	0.7456614	−3.7164151	−5.4236317
H	1.4474896	−4.5559331	−5.2712559
H	0.4093918	−3.7147242	−6.4678687
H	1.2681488	−2.7688590	−5.2057737

CH_3CN@2

N	−1.5139215	−1.9116658	−1.4884741
H	−0.9318602	−1.2322239	−1.0067085
C	−2.8641726	−1.7425193	−1.7178903
C	−3.3107380	−2.8924049	−2.3474084
H	−4.3277187	−3.0861618	−2.6710361
C	−2.1964362	−3.7721714	−2.4861227
H	−2.2098222	−4.7576421	−2.9387595

C	−1.0884057	−3.1463722	−1.9351904
C	0.3195603	−3.6498689	−1.6825445
N	1.7346753	−1.5445697	−1.6342683
H	1.3816768	−1.2252194	−0.7365591
C	1.3758571	−2.7255665	−2.2566352
C	2.2059129	−2.8559260	−3.3591370
H	2.1881059	−3.6723503	−4.0731427
C	3.0949872	−1.7415461	−3.3762867
H	3.8705331	−1.5490248	−4.1097200
C	2.7946664	−0.9432116	−2.2855757
C	3.4096593	0.3512379	−1.7829137
N	1.2969667	1.7273127	−1.8716801
H	0.7856983	1.0785129	−1.2804867
C	2.6062672	1.5407629	−2.2662393
C	2.9601809	2.6498354	−3.0162432
H	3.9254642	2.8219474	−3.4799653
C	1.8331521	3.5234090	−3.0581100
H	1.7822206	4.4806948	−3.5653249
C	0.8109417	2.9351837	−2.3289449
C	−0.5474604	3.4630196	−1.9094611
N	−1.9311158	1.3805202	−1.4718554
H	−1.4529983	1.1423266	−0.6079539
C	−1.6702784	2.4992484	−2.2412780
C	−2.6478001	2.5365389	−3.2231558
H	−2.7344378	3.2856637	−4.0028488
C	−3.5241289	1.4318403	−3.0155521
H	−4.3936528	1.1797538	−3.6130558
C	−3.0687641	0.7312469	−1.9114623
C	−3.5984135	−0.5137874	−1.2218175
C	0.4782586	−5.0441318	−2.3130192
H	−0.2418783	−5.7461782	−1.8712017
H	1.4927071	−5.4247706	−2.1342872
H	0.3046850	−5.0132991	−3.3967240
C	4.8569042	0.4657870	−2.3107285
H	4.8722842	0.4994872	−3.4084681
H	5.4448691	−0.3987945	−1.9779625
H	5.3242262	1.3814978	−1.9261761
C	−0.8108155	4.8027265	−2.6188036
H	−0.0406829	5.5361747	−2.3444066
H	−1.7914246	5.1982211	−2.3231164
H	−0.7973032	4.6848213	−3.7102731
C	−5.1053838	−0.6622447	−1.5310292

H	−5.6454798	0.2285771	−1.1868206
H	−5.5080249	−1.5431351	−1.0146754
H	−5.2744744	−0.7810682	−2.6095411
C	3.4657366	0.3489072	−0.2378930
C	3.2487171	1.5208682	0.5045945
C	3.2775886	1.5104031	1.8953454
C	3.5264986	0.3156204	2.5848856
C	3.7873576	−0.8569095	1.8654864
C	3.7623377	−0.8232382	0.4674207
C	−3.4446913	−0.3898689	0.3112327
C	−3.6292421	0.8443292	0.9589011
C	−3.4947623	0.9666951	2.3377831
C	−3.1882501	−0.1595395	3.1157624
C	−3.0349429	−1.4050871	2.4957649
C	−3.1640281	−1.5050023	1.1058987
C	0.0781319	0.1097411	1.7286679
N	0.0609650	−0.0059654	0.5731043
C	0.0887341	0.2444939	3.1698185
H	1.0801092	0.5690899	3.5150357
H	−0.6779668	0.9661705	3.4807751
H	−0.1579099	−0.7261160	3.6207216
C	−0.5212920	3.7214239	−0.3762126
H	0.2488115	4.4689953	−0.1401202
H	−1.4983164	4.0882896	−0.0297109
H	−0.2739735	2.8111195	0.1898695
C	0.5267705	−3.7843334	−0.1472348
H	−0.1989977	−4.5017797	0.2612296
H	1.5450833	−4.1351269	0.0743686
H	0.3719463	−2.8291553	0.3752021
H	−3.6297964	1.9275323	2.8358061
H	−2.8116509	−2.3003224	3.0743026
H	−3.8706150	1.7282983	0.3664296
H	−3.0314771	−2.4775011	0.6296970
H	3.0975280	2.4203912	2.4693312
H	4.0039287	−1.7957393	2.3723570
H	3.9549298	−1.7441908	−0.0857582
H	3.0381892	2.4539093	−0.0193733
C	−2.8492405	−1.0630543	5.3090947
H	−2.7980646	−0.6706745	6.3307223
H	−3.6951817	−1.7651131	5.2254503
H	−1.9133326	−1.6040906	5.0820211
C	3.7511131	−0.7874518	4.7034488

H	3.6606070	−0.5069398	5.7587800
H	3.0391723	−1.6000737	4.4775514
H	4.7766511	−1.1411179	4.5062065
O	−3.0265352	0.0709343	4.4623983
O	3.4541073	0.3937477	3.9583361

Cl^-@**3**

Cl	0.0000000	0.0000000	−1.6753013
N	−1.6578351	−1.6542811	0.5402057
H	−1.1596509	−1.1551543	−0.2150950
C	−1.2533411	−2.8640610	1.0619307
C	−2.2135354	−3.2487381	1.9871898
H	−2.1995912	−4.1570513	2.5806807
C	−3.2188759	−2.2388781	2.0100517
H	−4.1115483	−2.2346925	2.6269101
C	−2.8565545	−1.2593412	1.0940174
C	−3.5804131	0.0000000	0.6504195
N	−1.6578351	1.6542811	0.5402057
H	−1.1596509	1.1551543	−0.2150950
C	−2.8565545	1.2593412	1.0940174
C	−3.2188759	2.2388781	2.0100517
H	−4.1115483	2.2346925	2.6269101
C	−2.2135354	3.2487381	1.9871898
H	−2.1995912	4.1570513	2.5806807
C	−1.2533411	2.8640610	1.0619307
C	0.0000000	3.5698668	0.5765508
N	1.6578351	1.6542811	0.5402057
H	1.1596509	1.1551543	−0.2150950
C	1.2533411	2.8640610	1.0619307
C	2.2135354	3.2487381	1.9871898
H	2.1995912	4.1570513	2.5806807
C	3.2188759	2.2388781	2.0100517
H	4.1115483	2.2346925	2.6269101
C	2.8565545	1.2593412	1.0940174
C	3.5804131	0.0000000	0.6504195
N	1.6578351	−1.6542811	0.5402057
H	1.1596509	−1.1551543	−0.2150950
C	2.8565545	−1.2593412	1.0940174
C	3.2188759	−2.2388781	2.0100517
H	4.1115483	−2.2346925	2.6269101
C	2.2135354	−3.2487381	1.9871898

H	2.1995912	−4.1570513	2.5806807
C	1.2533411	−2.8640610	1.0619307
C	0.0000000	−3.5698668	0.5765508
C	−4.9943209	0.0000000	1.2580803
H	−5.5438976	−0.8932534	0.9303519
H	−5.5438976	0.8932534	0.9303519
H	−4.9616925	0.0000000	2.3562314
C	0.0000000	5.0171486	1.1210208
H	0.0000000	5.0215972	2.2197874
H	−0.8933434	5.5476642	0.7670292
H	0.8933434	5.5476642	0.7670292
C	4.9943209	0.0000000	1.2580803
H	5.5438976	0.8932534	0.9303519
H	5.5438976	−0.8932534	0.9303519
H	4.9616925	0.0000000	2.3562314
C	0.0000000	−5.0171486	1.1210208
H	0.8933434	−5.5476642	0.7670292
H	−0.8933434	−5.5476642	0.7670292
H	0.0000000	−5.0215972	2.2197874
C	0.0000000	3.6768737	−0.9644603
C	1.2026631	3.7578452	−1.6782776
H	2.1493375	3.6889976	−1.1401404
C	1.2142111	3.8981270	−3.0668211
C	0.0000000	3.9646266	−3.7469248
C	−1.2142111	3.8981270	−3.0668211
C	−1.2026631	3.7578452	−1.6782776
H	−2.1493375	3.6889976	−1.1401404
C	0.0000000	−3.6768737	−0.9644603
C	1.2026631	−3.7578452	−1.6782776
H	2.1493375	−3.6889976	−1.1401404
C	1.2142111	−3.8981270	−3.0668211
C	0.0000000	−3.9646266	−3.7469248
C	−1.2142111	−3.8981270	−3.0668211
C	−1.2026631	−3.7578452	−1.6782776
H	−2.1493375	−3.6889976	−1.1401404
C	−3.7202793	0.0000000	−0.8962040
H	−4.2710561	−0.8956235	−1.2204355
H	−4.2710561	0.8956235	−1.2204355
H	−2.7463109	0.0000000	−1.4046123
C	3.7202793	0.0000000	−0.8962040
H	4.2710561	0.8956235	−1.2204355
H	4.2710561	−0.8956235	−1.2204355

H	2.7463109	0.0000000	−1.4046123
H	2.1559992	3.9406561	−3.6127151
H	−2.1559992	3.9406561	−3.6127151
H	−2.1559992	−3.9406561	−3.6127151
H	2.1559992	−3.9406561	−3.6127151
Br	0.0000000	−4.1428373	−5.6620032
Br	0.0000000	4.1428373	−5.6620032

CH_3CN@3

Cl	0.0000000	0.0000000	−1.6753013
N	−1.6578351	−1.6542811	0.5402057
H	−1.1596509	−1.1551543	−0.2150950
C	−1.2533411	−2.8640610	1.0619307
C	−2.2135354	−3.2487381	1.9871898
H	−2.1995912	−4.1570513	2.5806807
C	−3.2188759	−2.2388781	2.0100517
H	−4.1115483	−2.2346925	2.6269101
C	−2.8565545	−1.2593412	1.0940174
C	−3.5804131	0.0000000	0.6504195
N	−1.6578351	1.6542811	0.5402057
H	−1.1596509	1.1551543	−0.2150950
C	−2.8565545	1.2593412	1.0940174
C	−3.2188759	2.2388781	2.0100517
H	−4.1115483	2.2346925	2.6269101
C	−2.2135354	3.2487381	1.9871898
H	−2.1995912	4.1570513	2.5806807
C	−1.2533411	2.8640610	1.0619307
C	0.0000000	3.5698668	0.5765508
N	1.6578351	1.6542811	0.5402057
H	1.1596509	1.1551543	−0.2150950
C	1.2533411	2.8640610	1.0619307
C	2.2135354	3.2487381	1.9871898
H	2.1995912	4.1570513	2.5806807
C	3.2188759	2.2388781	2.0100517
H	4.1115483	2.2346925	2.6269101
C	2.8565545	1.2593412	1.0940174
C	3.5804131	0.0000000	0.6504195
N	1.6578351	−1.6542811	0.5402057
H	1.1596509	−1.1551543	−0.2150950
C	2.8565545	−1.2593412	1.0940174
C	3.2188759	−2.2388781	2.0100517

H	4.1115483	−2.2346925	2.6269101
C	2.2135354	−3.2487381	1.9871898
H	2.1995912	−4.1570513	2.5806807
C	1.2533411	−2.8640610	1.0619307
C	0.0000000	−3.5698668	0.5765508
C	−4.9943209	0.0000000	1.2580803
H	−5.5438976	−0.8932534	0.9303519
H	−5.5438976	0.8932534	0.9303519
H	−4.9616925	0.0000000	2.3562314
C	0.0000000	5.0171486	1.1210208
H	0.0000000	5.0215972	2.2197874
H	−0.8933434	5.5476642	0.7670292
H	0.8933434	5.5476642	0.7670292
C	4.9943209	0.0000000	1.2580803
H	5.5438976	0.8932534	0.9303519
H	5.5438976	−0.8932534	0.9303519
H	4.9616925	0.0000000	2.3562314
C	0.0000000	−5.0171486	1.1210208
H	0.8933434	−5.5476642	0.7670292
H	−0.8933434	−5.5476642	0.7670292
H	0.0000000	−5.0215972	2.2197874
C	0.0000000	3.6768737	−0.9644603
C	1.2026631	3.7578452	−1.6782776
H	2.1493375	3.6889976	−1.1401404
C	1.2142111	3.8981270	−3.0668211
C	0.0000000	3.9646266	−3.7469248
C	−1.2142111	3.8981270	−3.0668211
C	−1.2026631	3.7578452	−1.6782776
H	−2.1493375	3.6889976	−1.1401404
C	0.0000000	−3.6768737	−0.9644603
C	1.2026631	−3.7578452	−1.6782776
H	2.1493375	−3.6889976	−1.1401404
C	1.2142111	−3.8981270	−3.0668211
C	0.0000000	−3.9646266	−3.7469248
C	−1.2142111	−3.8981270	−3.0668211
C	−1.2026631	−3.7578452	−1.6782776
H	−2.1493375	−3.6889976	−1.1401404
C	−3.7202793	0.0000000	−0.8962040
H	−4.2710561	−0.8956235	−1.2204355
H	−4.2710561	0.8956235	−1.2204355
H	−2.7463109	0.0000000	−1.4046123
C	3.7202793	0.0000000	−0.8962040

H	4.2710561	0.8956235	−1.2204355
H	4.2710561	−0.8956235	−1.2204355
H	2.7463109	0.0000000	−1.4046123
H	2.1559992	3.9406561	−3.6127151
H	−2.1559992	3.9406561	−3.6127151
H	−2.1559992	−3.9406561	−3.6127151
H	2.1559992	−3.9406561	−3.6127151
Br	0.0000000	−4.1428373	−5.6620032
Br	0.0000000	4.1428373	−5.6620032

Cl^-@**4**

Cl	0.0000000	0.0000000	−1.7034381
N	−1.6561209	−1.6614609	0.5105991
H	−1.1582828	−1.1679006	−0.2486156
C	−1.2522427	−2.8674215	1.0411842
C	−2.2109502	−3.2426030	1.9720737
H	−2.1970093	−4.1461349	2.5728849
C	−3.2147341	−2.2310620	1.9889951
H	−4.1057150	−2.2198924	2.6083379
C	−2.8526302	−1.2595678	1.0641936
C	−3.5741513	0.0000000	0.6165608
N	−1.6561209	1.6614609	0.5105991
H	−1.1582828	1.1679006	−0.2486156
C	−2.8526302	1.2595678	1.0641936
C	−3.2147341	2.2310620	1.9889951
H	−4.1057150	2.2198924	2.6083379
C	−2.2109502	3.2426030	1.9720737
H	−2.1970093	4.1461349	2.5728849
C	−1.2522427	2.8674215	1.0411842
C	0.0000000	3.5795512	0.5614706
N	1.6561209	1.6614609	0.5105991
H	1.1582828	1.1679006	−0.2486156
C	1.2522427	2.8674215	1.0411842
C	2.2109502	3.2426030	1.9720737
H	2.1970093	4.1461349	2.5728849
C	3.2147341	2.2310620	1.9889951
H	4.1057150	2.2198924	2.6083379
C	2.8526302	1.2595678	1.0641936
C	3.5741513	0.0000000	0.6165608
N	1.6561209	−1.6614609	0.5105991
H	1.1582828	−1.1679006	−0.2486156

C	2.8526302	−1.2595678	1.0641936
C	3.2147341	−2.2310620	1.9889951
H	4.1057150	−2.2198924	2.6083379
C	2.2109502	−3.2426030	1.9720737
H	2.1970093	−4.1461349	2.5728849
C	1.2522427	−2.8674215	1.0411842
C	0.0000000	−3.5795512	0.5614706
C	−4.9907512	0.0000000	1.2184411
H	−5.5386605	−0.8936174	0.8886235
H	−5.5386605	0.8936174	0.8886235
H	−4.9629095	0.0000000	2.3167424
C	0.0000000	5.0196651	1.1246986
H	0.0000000	5.0116387	2.2236199
H	−0.8932401	5.5541428	0.7763006
H	0.8932401	5.5541428	0.7763006
C	4.9907512	0.0000000	1.2184411
H	5.5386605	0.8936174	0.8886235
H	5.5386605	−0.8936174	0.8886235
H	4.9629095	0.0000000	2.3167424
C	0.0000000	−5.0196651	1.1246986
H	0.8932401	−5.5541428	0.7763006
H	−0.8932401	−5.5541428	0.7763006
H	0.0000000	−5.0116387	2.2236199
C	0.0000000	3.7082658	−0.9790063
C	1.2042152	3.8014161	−1.6901751
H	2.1484639	3.7239934	−1.1486175
C	1.2056463	3.9611980	−3.0770151
C	0.0000000	4.0369957	−3.7784044
C	−1.2056463	3.9611980	−3.0770151
C	−1.2042152	3.8014161	−1.6901751
H	−2.1484639	3.7239934	−1.1486175
C	0.0000000	−3.7082658	−0.9790063
C	1.2042152	−3.8014161	−1.6901751
H	2.1484639	−3.7239934	−1.1486175
C	1.2056463	−3.9611980	−3.0770151
C	0.0000000	−4.0369957	−3.7784044
C	−1.2056463	−3.9611980	−3.0770151
C	−1.2042152	−3.8014161	−1.6901751
H	−2.1484639	−3.7239934	−1.1486175
C	−3.7088594	0.0000000	−0.9302578
H	−4.2582016	−0.8960598	−1.2560349
H	−4.2582016	0.8960598	−1.2560349

H	−2.7331951	0.0000000	−1.4353221
C	3.7088594	0.0000000	−0.9302578
H	4.2582016	0.8960598	−1.2560349
H	4.2582016	−0.8960598	−1.2560349
H	2.7331951	0.0000000	−1.4353221
H	2.1552764	4.0089882	−3.6135627
H	−2.1552764	4.0089882	−3.6135627
H	−2.1552764	−4.0089882	−3.6135627
H	2.1552764	−4.0089882	−3.6135627
H	0.0000000	4.1388143	−4.8651674
H	0.0000000	−4.1388143	−4.8651674

CH_3CN@4

N	−1.5435781	−1.9017011	−1.5022785
H	−0.9594062	−1.2023631	−1.0527868
C	−2.8943892	−1.7437311	−1.7351518
C	−3.3395657	−2.9163198	−2.3228337
H	−4.3567713	−3.1244587	−2.6365961
C	−2.2235730	−3.7979594	−2.4319980
H	−2.2353724	−4.7989794	−2.8490763
C	−1.1164068	−3.1505050	−1.9048391
C	0.2903320	−3.6442606	−1.6262950
N	1.7146512	−1.5439252	−1.5527438
H	1.3642616	−1.2370378	−0.6504809
C	1.3486620	−2.7156926	−2.1894508
C	2.1673276	−2.8291565	−3.3019819
H	2.1416349	−3.6347081	−4.0279587
C	3.0559141	−1.7150745	−3.3117752
H	3.8232705	−1.5117520	−4.0508325
C	2.7669174	−0.9320996	−2.2067847
C	3.3828730	0.3615946	−1.7056082
N	1.2701342	1.7392155	−1.8410454
H	0.7347263	1.0782185	−1.2857413
C	2.5901907	1.5538311	−2.1988382
C	2.9643585	2.6636691	−2.9380586
H	3.9426823	2.8382221	−3.3724188
C	1.8377589	3.5345679	−3.0148128
H	1.8006393	4.4914264	−3.5239449
C	0.7943891	2.9443096	−2.3181172
C	−0.5801049	3.4702100	−1.9506886
N	−1.9688288	1.3926101	−1.5145957

H	−1.5131396	1.1725627	−0.6342915
C	−1.6876179	2.4946652	−2.2998445
C	−2.6346048	2.5060621	−3.3121396
H	−2.7004896	3.2378589	−4.1101016
C	−3.5129499	1.4023336	−3.1072418
H	−4.3625823	1.1331761	−3.7255899
C	−3.0891636	0.7282569	−1.9742431
C	−3.6326994	−0.5046233	−1.2737610
C	0.4686742	−5.0413211	−2.2458526
H	−0.2503237	−5.7470657	−1.8080948
H	1.4840499	−5.4117630	−2.0521977
H	0.3072520	−5.0192671	−3.3316235
C	4.8334402	0.4666784	−2.2321483
H	4.8472234	0.5004229	−3.3296155
H	5.4153174	−0.4018856	−1.8996146
H	5.3070046	1.3791324	−1.8477511
C	−0.8294054	4.7958215	−2.6911345
H	−0.0736219	5.5397233	−2.4054508
H	−1.8215742	5.1892393	−2.4334866
H	−0.7806066	4.6591185	−3.7793500
C	−5.1353408	−0.6555448	−1.6052863
H	−5.6788719	0.2444368	−1.2919587
H	−5.5509112	−1.5234060	−1.0774310
H	−5.2829706	−0.7982915	−2.6839457
C	3.4641123	0.3694905	−0.1610087
C	3.2808299	1.5531672	0.5682459
H	3.0327600	2.4724128	0.0360257
C	3.4198287	1.5667715	1.9589873
C	3.7373242	0.3928246	2.6485185
C	3.9206537	−0.7940296	1.9321745
C	3.7929237	−0.8012284	0.5413764
H	3.9469121	−1.7287985	−0.0128699
C	−3.5155649	−0.3525098	0.2617680
C	−3.7484891	0.8887579	0.8754386
H	−3.9897125	1.7530437	0.2543321
C	−3.6654260	1.0322413	2.2622270
C	−3.3602781	−0.0707568	3.0657980
C	−3.1465488	−1.3154459	2.4675098
C	−3.2239501	−1.4536134	1.0792571
H	−3.0525841	−2.4271324	0.6183448
C	0.0118379	0.1516142	1.6745336
N	−0.0373332	0.0223200	0.5224301

C	0.0870153	0.3181178	3.1091869
H	1.1218442	0.5621383	3.3859248
H	−0.5923375	1.1233203	3.4179571
H	−0.2268997	−0.6108247	3.6027412
H	−3.3014255	0.0376309	4.1503775
H	3.8540977	0.4038384	3.7338709
C	−0.6031003	3.7552691	−0.4221523
H	0.1540224	4.5127843	−0.1758429
H	−1.5929535	4.1198915	−0.1115383
H	−0.3659988	2.8561381	0.1656779
C	0.4703560	−3.7678710	−0.0869844
H	−0.2554116	−4.4904089	0.3117908
H	1.4875539	−4.1059607	0.1579182
H	0.2918925	−2.8114228	0.4260173
H	3.2852159	2.5029821	2.5044261
H	4.1783180	−1.7159784	2.4560354
H	−2.9234886	−2.1884143	3.0838163
H	−3.8504255	2.0072497	2.7165604

Cl^-@**5**

Cl	−0.0000000	0.0000000	−1.4045454
N	−1.8407828	−1.4443891	0.8198575
H	−1.2859333	−1.0077778	0.0657376
C	−1.5850333	−2.6952241	1.3386806
C	−2.5882591	−2.9667636	2.2581356
H	−2.6849955	−3.8727285	2.8476721
C	−3.4674192	−1.8453441	2.2799254
H	−4.3556531	−1.7372236	2.8934776
C	−2.9875962	−0.9127644	1.3691696
C	−3.5555605	0.4249411	0.9279452
N	−1.4485254	1.8356409	0.8215457
H	−1.0132319	1.2815337	0.0661000
C	−2.6868824	1.5880612	1.3730856
C	−2.9317528	2.6054043	2.2865877
H	−3.8200476	2.7091964	2.9011468
C	−1.8130477	3.4879624	2.2642304
H	−1.6919281	4.3893356	2.8559207
C	−0.9040989	2.9900123	1.3415943
C	0.4230989	3.5428273	0.8535765
N	1.8407828	1.4443891	0.8198575
H	1.2859333	1.0077778	0.0657376

C	1.5850333	2.6952241	1.3386806
C	2.5882591	2.9667636	2.2581356
H	2.6849955	3.8727285	2.8476721
C	3.4674192	1.8453441	2.2799254
H	4.3556531	1.7372236	2.8934776
C	2.9875962	0.9127644	1.3691696
C	3.5555605	−0.4249411	0.9279452
N	1.4485254	−1.8356409	0.8215457
H	1.0132319	−1.2815337	0.0661000
C	2.6868824	−1.5880612	1.3730856
C	2.9317528	−2.6054043	2.2865877
H	3.8200476	−2.7091964	2.9011468
C	1.8130477	−3.4879624	2.2642304
H	1.6919281	−4.3893356	2.8559207
C	0.9040989	−2.9900123	1.3415943
C	−0.4230989	−3.5428273	0.8535765
C	−4.9594729	0.5931052	1.5359286
H	−5.6123355	−0.2271413	1.2069703
H	−5.3978143	1.5462099	1.2093591
H	−4.9269054	0.5874766	2.6341337
C	0.5936879	4.9811295	1.3956306
H	0.5943155	4.9880472	2.4945509
H	−0.2308887	5.6130759	1.0408330
H	1.5430206	5.4014148	1.0398874
C	4.9594729	−0.5931052	1.5359286
H	5.6123355	0.2271413	1.2069703
H	5.3978143	−1.5462099	1.2093591
H	4.9269054	−0.5874766	2.6341337
C	−0.5936879	−4.9811295	1.3956306
H	0.2308887	−5.6130759	1.0408330
H	−1.5430206	−5.4014148	1.0398874
H	−0.5943155	−4.9880472	2.4945509
C	0.4329586	3.6415562	−0.6875446
C	1.6335687	3.5703147	−1.4075138
H	2.5668971	3.3968458	−0.8704766
C	1.6516522	3.6853909	−2.7948102
C	0.4541959	3.8815954	−3.4912803
C	−0.7545300	3.9759729	−2.7885288
C	−0.7541117	3.8584280	−1.4010454
H	−1.6988245	3.9128365	−0.8579651
C	−0.4329586	−3.6415562	−0.6875446
C	0.7541117	−3.8584280	−1.4010454

H	1.6988245	−3.9128365	−0.8579651
C	0.7545300	−3.9759729	−2.7885288
C	−0.4541959	−3.8815954	−3.4912803
C	−1.6516522	−3.6853909	−2.7948102
C	−1.6335687	−3.5703147	−1.4075138
H	−2.5668971	−3.3968458	−0.8704766
C	−3.6941324	0.4442048	−0.6187992
H	−4.3513645	−0.3762421	−0.9433892
H	−4.1290374	1.4014571	−0.9427071
H	−2.7271088	0.3245667	−1.1264991
C	3.6941324	−0.4442048	−0.6187992
H	4.3513645	0.3762421	−0.9433892
H	4.1290374	−1.4014571	−0.9427071
H	2.7271088	−0.3245667	−1.1264991
H	2.5850226	3.6059095	−3.3526922
H	−1.6948715	4.1215630	−3.3236162
H	−2.5850226	−3.6059095	−3.3526922
H	1.6948715	−4.1215630	−3.3236162
N	0.5486307	3.9713832	−4.9095591
N	−0.5486307	−3.9713832	−4.9095591
N	−0.4695982	4.1326032	−5.5805622
N	0.4695982	−4.1326032	−5.5805622
N	−1.3323420	4.2855567	−6.3224209
N	1.3323420	−4.2855567	−6.3224209

CH_3CN@5

N	−1.0981357	1.6981668	−1.6133666
H	−0.5561460	1.1009535	−0.9947561
C	−1.4215626	1.3701746	−2.9145861
C	−2.1008549	2.4541859	−3.4450178
H	−2.5009822	2.5248187	−4.4505823
C	−2.1749567	3.4568850	−2.4333914
H	−2.6455481	4.4293328	−2.5278997
C	−1.5351455	2.9702908	−1.3036670
C	−1.1984886	3.6342426	0.0171790
N	−1.0100185	1.7098015	1.6612360
H	−0.1209092	1.3786925	1.2972938
C	−1.6864584	2.8256517	1.2039408
C	−2.7518642	3.0210295	2.0686033
H	−3.4916601	3.8107690	1.9947036
C	−2.6912803	2.0166668	3.0781612

H	−3.3803141	1.8956613	3.9069259
C	−1.5915293	1.2177773	2.8154034
C	−1.0209428	0.0193401	3.5522468
N	−1.2305443	−1.5920062	1.6221877
H	−0.7768825	−0.9611600	0.9689170
C	−1.5105131	−1.2737177	2.9357802
C	−2.1370645	−2.3773560	3.4900960
H	−2.4877064	−2.4625014	4.5128341
C	−2.2245502	−3.3805567	2.4798671
H	−2.6613309	−4.3668891	2.5916992
C	−1.6456202	−2.8744787	1.3260946
C	−1.3173742	−3.5374566	0.0026120
N	−1.0361714	−1.6246969	−1.6427517
H	−0.1322388	−1.3367463	−1.2814036
C	−1.7641385	−2.7089848	−1.1856855
C	−2.8321419	−2.8587495	−2.0555965
H	−3.6072066	−3.6141201	−1.9828439
C	−2.7208913	−1.8614579	−3.0677877
H	−3.4004734	−1.7115441	−3.8994848
C	−1.5880487	−1.1115093	−2.8020935
C	−0.9678084	0.0653808	−3.5342026
C	−1.8456645	5.0293199	0.0613812
H	−1.4644569	5.6516554	−0.7594910
H	−1.6102079	5.5236605	1.0131605
H	−2.9372638	4.9656784	−0.0363857
C	−1.4590874	0.0746845	5.0333123
H	−2.5520189	0.0100851	5.1181351
H	−1.1261803	1.0164197	5.4864937
H	−1.0150838	−0.7618377	5.5878095
C	−2.0024759	−4.9139344	−0.0593618
H	−1.6437550	−5.5555800	0.7567025
H	−1.7750042	−5.4055065	−1.0145343
H	−3.0920953	−4.8198566	0.0339671
C	−1.4030487	0.0285457	−5.0170611
H	−1.0942108	−0.9196444	−5.4741281
H	−0.9362223	0.8567906	−5.5650335
H	−2.4936013	0.1216669	−5.1050769
C	0.5236387	0.0623951	3.5197645
C	1.2849511	−1.1063338	3.3782715
H	0.7763125	−2.0669547	3.2887190
C	2.6766433	−1.0607320	3.3341027
C	3.3381707	0.1696416	3.4315085

C	2.5969225	1.3473392	3.6088481
C	1.2063517	1.2814831	3.6588602
H	0.6337300	2.2017963	3.7854366
C	0.5731215	−0.0338612	−3.5026345
C	1.2124780	−1.2778652	−3.6267594
H	0.6080193	−2.1802245	−3.7326542
C	2.6011763	−1.3889476	−3.5986597
C	3.3850498	−0.2333260	−3.4594265
C	2.7663160	1.0192993	−3.3683332
C	1.3762884	1.1102119	−3.3915099
H	0.9009927	2.0883834	−3.3105798
C	0.3434700	3.8106252	0.1047707
H	0.6894413	4.4481328	−0.7207537
H	0.6235845	4.2774763	1.0602002
H	0.8761167	2.8521308	0.0217913
C	0.2211342	−3.7558420	−0.0642104
H	0.5360524	−4.4148801	0.7566155
H	0.5061850	−4.2134519	−1.0228111
H	0.7729526	−2.8109958	0.0483512
H	3.2630316	−1.9729629	3.2173077
H	3.1013887	2.3107950	3.7023026
H	3.3842650	1.9128612	−3.2745175
H	3.0718215	−2.3697493	−3.6898468
N	1.1180611	0.0528008	0.0087919
C	2.2521791	−0.1924605	−0.0405643
C	3.6634322	−0.5085609	−0.1096963
H	4.1084115	−0.4692219	0.8940559
H	3.7885663	−1.5185617	−0.5234312
H	4.1725225	0.2036097	−0.7728780
N	4.7555728	0.1362842	3.3072885
N	4.8048113	−0.2470679	−3.3742551
N	5.3929630	1.1895943	3.4184964
N	5.3977238	−1.3289822	−3.4362584
N	6.0990703	2.0866577	3.4964438
N	6.0649595	−2.2585725	−3.4727009

Cl^-@**6**

C	1.2535077	−2.8552012	−1.4221729
N	1.6604766	−1.6296378	−0.9374321
H	1.1696890	−1.1143665	−0.1906859
C	2.2125484	−3.2706639	−2.3334803

H	2.1973583	−4.1967706	−2.8987077
C	3.2206633	−2.2638311	−2.3854424
H	4.1161356	−2.2831058	−2.9975525
C	2.8629701	−1.2574409	−1.4980384
N	1.6604766	1.6296378	−0.9374321
H	1.1696890	1.1143665	−0.1906859
C	3.5954516	0.0000000	−1.0664393
C	3.7485242	0.0000000	0.4802275
N	−1.6604766	1.6296378	−0.9374321
H	−1.1696890	1.1143665	−0.1906859
N	−1.6604766	−1.6296378	−0.9374321
H	−1.1696890	−1.1143665	−0.1906859
C	5.0008783	0.0000000	−1.6913244
H	4.9541177	0.0000000	−2.7891736
H	5.5547212	−0.8920946	−1.3688444
H	5.5547212	0.8920946	−1.3688444
C	2.8629701	1.2574409	−1.4980384
C	3.2206633	2.2638311	−2.3854424
H	4.1161356	2.2831058	−2.9975525
C	2.2125484	3.2706639	−2.3334803
H	2.1973583	4.1967706	−2.8987077
C	1.2535077	2.8552012	−1.4221729
C	0.0000000	3.5359028	−0.9071283
C	0.0000000	3.5318327	0.6376876
C	1.2037536	3.5297988	1.3514108
C	1.1825633	3.4771522	2.7440840
C	0.0000000	3.4330623	3.4739745
C	−1.1825633	3.4771522	2.7440840
C	−1.2037536	3.5297988	1.3514108
C	0.0000000	5.0132976	−1.3583931
H	0.0000000	5.0802565	−2.4545667
H	0.8946245	5.5218647	−0.9767515
H	−0.8946245	5.5218647	−0.9767515
C	−1.2535077	2.8552012	−1.4221729
C	−2.2125484	3.2706639	−2.3334803
H	−2.1973583	4.1967706	−2.8987077
C	−3.2206633	2.2638311	−2.3854424
H	−4.1161356	2.2831058	−2.9975525
C	−2.8629701	1.2574409	−1.4980384
C	−3.5954516	0.0000000	−1.0664393
C	−3.7485242	0.0000000	0.4802275
C	−5.0008783	0.0000000	−1.6913244

H	−4.9541177	0.0000000	−2.7891736
H	−5.5547212	0.8920946	−1.3688444
H	−5.5547212	−0.8920946	−1.3688444
C	−2.8629701	−1.2574409	−1.4980384
C	−3.2206633	−2.2638311	−2.3854424
H	−4.1161356	−2.2831058	−2.9975525
C	−2.2125484	−3.2706639	−2.3334803
H	−2.1973583	−4.1967706	−2.8987077
C	−1.2535077	−2.8552012	−1.4221729
C	0.0000000	−3.5359028	−0.9071283
C	0.0000000	−3.5318327	0.6376876
C	−1.2037536	−3.5297988	1.3514108
C	−1.1825633	−3.4771522	2.7440840
C	0.0000000	−3.4330623	3.4739745
C	1.1825633	−3.4771522	2.7440840
C	1.2037536	−3.5297988	1.3514108
C	0.0000000	−5.0132976	−1.3583931
H	0.0000000	−5.0802565	−2.4545667
H	−0.8946245	−5.5218647	−0.9767515
H	0.8946245	−5.5218647	−0.9767515
H	2.7773616	0.0000000	0.9941427
H	4.2973868	−0.8961369	0.8038148
H	4.2973868	0.8961369	0.8038148
H	−4.2973868	−0.8961369	0.8038148
H	−2.7773616	0.0000000	0.9941427
H	−4.2973868	0.8961369	0.8038148
H	0.0000000	−3.3639028	4.5579822
H	2.1597287	−3.5379840	0.8304279
H	−2.1597287	−3.5379840	0.8304279
H	0.0000000	3.3639028	4.5579822
H	−2.1597287	3.5379840	0.8304279
H	2.1597287	3.5379840	0.8304279
N	−2.4708694	3.4550441	3.4806053
N	2.4708694	3.4550441	3.4806053
N	2.4708694	−3.4550441	3.4806053
N	−2.4708694	−3.4550441	3.4806053
O	−3.5113447	3.4717949	2.8159199
O	−2.4264398	3.4304100	4.7146977
O	3.5113447	3.4717949	2.8159199
O	2.4264398	3.4304100	4.7146977
O	3.5113447	−3.4717949	2.8159199
O	2.4264398	−3.4304100	4.7146977

O	−2.4264398	−3.4304100	4.7146977
O	−3.5113447	−3.4717949	2.8159199
Cl	0.0000000	0.0000000	1.3186123

CH_3CN@6

N	−1.5456908	−1.8985455	−1.5331303
H	−0.9691273	−1.1863301	−1.0968166
C	−2.8853678	−1.7459565	−1.8358371
C	−3.3108212	−2.9437990	−2.3841732
H	−4.3163050	−3.1663392	−2.7246066
C	−2.1954266	−3.8332062	−2.4025117
H	−2.1949313	−4.8531304	−2.7703369
C	−1.1106218	−3.1678757	−1.8545459
C	0.2850267	−3.6401632	−1.4996473
N	1.7032215	−1.5349659	−1.5522362
H	1.3681026	−1.1984199	−0.6539036
C	1.3423250	−2.7457393	−2.1164524
C	2.1429167	−2.9141864	−3.2340617
H	2.1121314	−3.7589150	−3.9132662
C	3.0229127	−1.7972305	−3.3208515
H	3.7807400	−1.6341894	−4.0794999
C	2.7447803	−0.9548046	−2.2595419
C	3.3851061	0.3494696	−1.8246361
N	1.2726008	1.7249455	−1.8705821
H	0.7393280	1.0388203	−1.3467243
C	2.5799892	1.5454335	−2.2804582
C	2.9461762	2.6918791	−2.9645447
H	3.9154975	2.8845047	−3.4111090
C	1.8275246	3.5764653	−2.9594035
H	1.7882305	4.5614093	−3.4109324
C	0.7991924	2.9610983	−2.2640441
C	−0.5606253	3.4734900	−1.8316057
N	−1.9485106	1.3714823	−1.5241048
H	−1.4861270	1.1076473	−0.6584477
C	−1.6746658	2.5269789	−2.2339116
C	−2.6263131	2.6080287	−3.2370399
H	−2.6962509	3.3931985	−3.9818989
C	−3.5070506	1.4959575	−3.1028550
H	−4.3645390	1.2759989	−3.7293450
C	−3.0784950	0.7435442	−2.0241108
C	−3.6454297	−0.5152780	−1.3951853

C	0.4802567	−5.0789001	−2.0083498
H	−0.2470268	−5.7506623	−1.5331616
H	1.4909357	−5.4293307	−1.7630347
H	0.3437677	−5.1416484	−3.0960887
C	4.8159540	0.4392023	−2.4021637
H	4.7828001	0.4668932	−3.4987408
H	5.4052543	−0.4322818	−2.0905360
H	5.3123894	1.3505111	−2.0449086
C	−0.8127372	4.8486660	−2.4742491
H	−0.0483772	5.5662196	−2.1475216
H	−1.7972770	5.2286362	−2.1726436
H	−0.7812014	4.7898828	−3.5701561
C	−5.1359501	−0.6583078	−1.7747904
H	−5.6916740	0.2362292	−1.4669924
H	−5.5694699	−1.5361075	−1.2793802
H	−5.2425962	−0.7822970	−2.8601251
C	3.5048408	0.3671675	−0.2850036
C	3.2530874	1.5321290	0.4481138
H	2.9986918	2.4639931	−0.0547979
C	3.2892233	1.4889438	1.8414480
C	3.5964743	0.3335853	2.5522399
C	3.8906769	−0.7989736	1.7994569
C	3.8533085	−0.8020047	0.4064659
H	4.0673216	−1.7248457	−0.1317187
C	−3.5596263	−0.4001055	0.1437030
C	−3.7552869	0.8359510	0.7744100
H	−4.0250257	1.7205075	0.1983534
C	−3.5368413	0.9523156	2.1456953
C	−3.1568028	−0.1254525	2.9384317
C	−3.0267426	−1.3520805	2.2984461
C	−3.2323264	−1.5120275	0.9295636
H	−3.0907577	−2.4924710	0.4763972
C	−0.0087304	0.2554267	1.8334692
N	−0.0205407	0.0549520	0.6877069
C	−0.0054506	0.5077875	3.2592922
H	0.9941024	0.8264335	3.5879049
H	−0.7255855	1.3056267	3.4957945
H	−0.3028366	−0.4063437	3.7949504
H	−2.9578657	−0.0104456	4.0003945
H	3.6096660	0.3116484	3.6389647
N	−2.5545019	−2.5150336	3.0896676
O	−2.0000523	−2.2719371	4.1697422

O	−2.7193162	−3.6367133	2.6137409
N	−3.6213017	2.2937801	2.7731729
O	−4.2297760	3.1735409	2.1656012
O	−3.0464015	2.4416880	3.8590924
N	4.2282563	−2.0602021	2.5165182
O	4.4823556	−3.0516566	1.8301480
O	4.2232558	−2.0291517	3.7490861
N	2.9301003	2.7130302	2.5993325
O	2.6250014	2.5701387	3.7912390
O	2.9377783	3.7845048	1.9951806
C	−0.5558675	3.6510160	−0.2868500
H	0.2153349	4.3774318	0.0026162
H	−1.5345736	4.0075840	0.0646769
H	−0.3225733	2.7106745	0.2334309
C	0.4285579	−3.6439317	0.0480790
H	−0.3112084	−4.3262194	0.4885282
H	1.4363683	−3.9678640	0.3436454
H	0.2452749	−2.6499031	0.4835134

Cl^-@**7**

Cl	−0.0003419	−0.0001030	−1.7092366
N	0.9125416	−2.1392146	0.5745466
H	0.6547781	−1.4585305	−0.1549415
C	2.1547322	−2.2375658	1.1630991
C	2.1171334	−3.3452122	1.9997208
H	2.9314710	−3.6992963	2.6230318
C	0.8248233	−3.9349477	1.8850426
H	0.4734819	−4.8227093	2.4005130
C	0.1006127	−3.1728798	0.9823540
C	−1.3173226	−3.3288253	0.4666126
N	−2.1359051	−0.9364803	0.5545393
H	−1.5441558	−0.6420720	−0.2363544
C	−2.1656589	−2.2157292	1.0596258
C	−3.0813852	−2.2276953	2.0989828
H	−3.3495176	−3.0866873	2.7052699
C	−3.6250660	−0.9145092	2.2021558
H	−4.3809758	−0.5866782	2.9080574
C	−3.0318265	−0.1290920	1.2223977
C	−3.2955848	1.3046777	0.7993051
N	−0.9127652	2.1386322	0.5749226
H	−0.6548422	1.4575787	−0.1541238

C	−2.1549872	2.2372429	1.1633659
C	−2.1175228	3.3454539	1.9992474
H	−2.9319286	3.6998727	2.6222801
C	−0.8252886	3.9352760	1.8841944
H	−0.4740195	4.8233283	2.3992034
C	−0.1010298	3.1727560	0.9819157
C	1.3168595	3.3285344	0.4661071
N	2.1356613	0.9361790	0.5534940
H	1.5439372	0.6419273	−0.2374490
C	2.1651964	2.2153046	1.0588958
C	3.0809958	2.2272247	2.0981915
H	3.3491398	3.0861751	2.7045348
C	3.6247371	0.9140541	2.2011863
H	4.3806823	0.5861768	2.9070308
C	3.0314945	0.1287126	1.2213766
C	3.2952348	−1.3051214	0.7984599
C	−1.8795420	−4.6982492	0.9058467
H	−1.2647924	−5.5060259	0.4882372
H	−2.9139359	−4.8071010	0.5562387
H	−1.8697250	−4.7825592	2.0004846
C	−4.5810879	1.7956512	1.4887829
H	−4.4765312	1.7937747	2.5825181
H	−5.4217339	1.1418356	1.2202960
H	−4.8126985	2.8202028	1.1668584
C	1.8792326	4.6978660	0.9054898
H	1.2646187	5.5057641	0.4879251
H	2.9136613	4.8066382	0.5559881
H	1.8693302	4.7820454	2.0001338
C	4.5809301	−1.7959068	1.4877369
H	5.4214852	−1.1421111	1.2189251
H	4.8124864	−2.8205309	1.1660017
H	4.4766467	−1.7938019	2.5814937
C	−1.3789231	−3.2821650	−1.0843641
C	−2.5526151	−2.9053303	−1.7613351
C	−2.6168812	−2.7530993	−3.1463841
C	−1.4976341	−3.0302838	−3.9269032
C	−0.3395366	−3.4841830	−3.3041548
C	−0.2993615	−3.6241780	−1.9149649
C	1.3787457	3.2821176	−1.0848861
C	0.2991435	3.6227585	−1.9159762
C	0.3404270	3.4835702	−3.3052384
C	1.4997259	3.0321677	−3.9275447

C	2.6187865	2.7558798	−3.1464148
C	2.5533974	2.9073007	−1.7613404
F	0.8475011	−4.1257426	−1.4166221
F	−3.7044654	−2.6855443	−1.0934960
F	−3.7643708	−2.3616183	−3.7362495
F	−1.5479982	−2.8964007	−5.2643989
F	0.7375830	−3.8022696	−4.0475809
F	−0.8489414	4.1220435	−1.4182461
F	−0.7367372	3.8003696	−4.0491415
F	1.5513610	2.8996548	−5.2651425
F	3.7671772	2.3662254	−3.7357881
F	3.7051400	2.6889251	−1.0928532
C	−3.5209179	1.3537667	−0.7375316
H	−4.3550247	0.6929955	−1.0159758
H	−3.7532795	2.3830725	−1.0467512
H	−2.6325241	1.0308568	−1.2982659
C	3.5201640	−1.3545570	−0.7383807
H	4.3542468	−0.6939090	−1.0171809
H	3.7524103	−2.3839424	−1.0474179
H	2.6316682	−1.0317308	−1.2990059

CH_3CN@7

N	−1.5319312	−1.9137339	−1.5027833
H	−0.9256758	−1.1514532	−1.2171355
C	−2.9000335	−1.8152582	−1.6422891
C	−3.3521645	−3.0443092	−2.0900232
H	−4.3832852	−3.3085080	−2.2977457
C	−2.2211384	−3.9030570	−2.2041186
H	−2.2308024	−4.9376594	−2.5288382
C	−1.0993355	−3.1818841	−1.8245093
C	0.3408814	−3.6054915	−1.6197194
N	1.7549582	−1.4983608	−1.5629882
H	1.5051680	−1.2665298	−0.6069162
C	1.3118798	−2.6209802	−2.2413329
C	1.9711102	−2.6269021	−3.4596832
H	1.8488437	−3.3694333	−4.2408338
C	2.8500115	−1.5062297	−3.4946269
H	3.5167685	−1.2354572	−4.3059970
C	2.7145717	−0.8283530	−2.2971312
C	3.4201088	0.4121671	−1.7687319
N	1.2911157	1.7564422	−1.8764784

H	0.7351411	1.0376243	−1.4243765
C	2.6305494	1.6336165	−2.1779231
C	3.0133339	2.8030364	−2.8114962
H	4.0072473	3.0342487	−3.1786482
C	1.8716985	3.6534224	−2.8705719
H	1.8330067	4.6469750	−3.3034954
C	0.8132217	2.9863247	−2.2728102
C	−0.5908403	3.4390100	−1.9282360
N	−1.9765933	1.3600241	−1.4742380
H	−1.6174661	1.2356495	−0.5334444
C	−1.6260609	2.4013255	−2.3165057
C	−2.4261395	2.2808700	−3.4407550
H	−2.4033231	2.9348635	−4.3058028
C	−3.2953412	1.1684937	−3.2502795
H	−4.0528291	0.8165389	−3.9421380
C	−3.0126741	0.6213690	−2.0124040
C	−3.6420261	−0.5514754	−1.2748679
C	0.5614873	−4.9929023	−2.2492202
H	−0.0894761	−5.7371568	−1.7705232
H	1.6055584	−5.3040752	−2.1143418
H	0.3354660	−4.9839032	−3.3234068
C	4.8431198	0.5020130	−2.3684885
H	4.7816022	0.6097241	−3.4583680
H	5.4146681	−0.4030502	−2.1365343
H	5.3688256	1.3761067	−1.9632431
C	−0.9001130	4.7560196	−2.6629762
H	−0.1967294	5.5401980	−2.3516537
H	−1.9196877	5.0867169	−2.4264555
H	−0.8151239	4.6348171	−3.7505841
C	−5.1258422	−0.6961162	−1.6895923
H	−5.6713755	0.2330827	−1.4930803
H	−5.5948074	−1.5164399	−1.1316917
H	−5.1921786	−0.9234336	−2.7606399
C	3.5631741	0.2886689	−0.2247361
C	3.2360615	1.2916226	0.7031398
C	3.2983800	1.0810586	2.0853719
C	3.7233512	−0.1404936	2.5973485
C	4.1122302	−1.1447728	1.7113803
C	4.0413943	−0.9118669	0.3373824
C	−3.6086437	−0.2626753	0.2532497
C	−4.0314181	0.9924457	0.7347469
C	−3.9617662	1.3662976	2.0776462

C	−3.4834637	0.4549241	3.0186208
C	−3.1058721	−0.8150487	2.5941091
C	−3.1793858	−1.1653342	1.2406490
C	0.0370780	0.1610153	1.8414891
N	0.0452934	0.0532403	0.6845095
C	0.0207712	0.2920979	3.2855756
H	1.0385410	0.4586381	3.6623829
H	−0.6076028	1.1455507	3.5741370
H	−0.3929890	−0.6201858	3.7355549
C	−0.6564178	3.7043383	−0.3985188
H	0.0405594	4.5108403	−0.1346922
H	−1.6734217	3.9929767	−0.0951622
H	−0.3502161	2.8204251	0.1813889
C	0.6036776	−3.7129702	−0.0918832
H	−0.0511713	−4.4813186	0.3400955
H	1.6520516	−3.9787117	0.1063592
H	0.3734093	−2.7711334	0.4287870
F	−3.3849484	0.7973534	4.3143113
F	−4.3535341	2.5896331	2.4693625
F	−2.6406917	−1.7045598	3.4952376
F	−2.8011403	−2.4224095	0.9589504
F	−4.5316162	1.9137707	−0.1094174
F	2.8297287	2.5174695	0.3346430
F	2.9187018	2.0592741	2.9340175
F	3.7553290	−0.3460896	3.9253147
F	4.5524906	−2.3218482	2.1837029
F	4.4539428	−1.9202636	−0.4540508

Cl^-@**8**

Cl	0.0000000	0.0000000	−1.4235726
N	−1.6611158	−1.6489847	0.7932174
H	−1.1629705	−1.1476615	0.0397860
C	−1.2546463	−2.8579690	1.3157735
C	−2.2161750	−3.2464230	2.2378608
H	−2.2021498	−4.1551496	2.8305963
C	−3.2237357	−2.2392711	2.2591177
H	−4.1173679	−2.2378814	2.8743846
C	−2.8623874	−1.2581857	1.3445182
C	−3.5877712	0.0000000	0.9010530
N	−1.6611158	1.6489847	0.7932174
H	−1.1629705	1.1476615	0.0397860

C	−2.8623874	1.2581857	1.3445182
C	−3.2237357	2.2392711	2.2591177
H	−4.1173679	2.2378814	2.8743846
C	−2.2161750	3.2464230	2.2378608
H	−2.2021498	4.1551496	2.8305963
C	−1.2546463	2.8579690	1.3157735
C	0.0000000	3.5611564	0.8322310
N	1.6611158	1.6489847	0.7932174
H	1.1629705	1.1476615	0.0397860
C	1.2546463	2.8579690	1.3157735
C	2.2161750	3.2464230	2.2378608
H	2.2021498	4.1551496	2.8305963
C	3.2237357	2.2392711	2.2591177
H	4.1173679	2.2378814	2.8743846
C	2.8623874	1.2581857	1.3445182
C	3.5877712	0.0000000	0.9010530
N	1.6611158	−1.6489847	0.7932174
H	1.1629705	−1.1476615	0.0397860
C	2.8623874	−1.2581857	1.3445182
C	3.2237357	−2.2392711	2.2591177
H	4.1173679	−2.2378814	2.8743846
C	2.2161750	−3.2464230	2.2378608
H	2.2021498	−4.1551496	2.8305963
C	1.2546463	−2.8579690	1.3157735
C	0.0000000	−3.5611564	0.8322310
C	−5.0009699	0.0000000	1.5101324
H	−5.5510432	−0.8928890	1.1826014
H	−5.5510432	0.8928890	1.1826014
H	−4.9675948	0.0000000	2.6082208
C	0.0000000	5.0109740	1.3738163
H	0.0000000	5.0113099	2.4722730
H	−0.8938115	5.5416053	1.0216752
H	0.8938115	5.5416053	1.0216752
C	5.0009699	0.0000000	1.5101324
H	5.5510432	0.8928890	1.1826014
H	5.5510432	−0.8928890	1.1826014
H	4.9675948	0.0000000	2.6082208
C	0.0000000	−5.0109740	1.3738163
H	0.8938115	−5.5416053	1.0216752
H	−0.8938115	−5.5416053	1.0216752
H	0.0000000	−5.0113099	2.4722730
C	0.0000000	3.6790168	−0.7057536

C	1.2077102	3.7672005	−1.4166929
H	2.1507092	3.6908897	−0.8744013
C	1.2174871	3.9289374	−2.7979037
C	0.0000000	4.0089268	−3.4764978
C	−1.2174871	3.9289374	−2.7979037
C	−1.2077102	3.7672005	−1.4166929
H	−2.1507092	3.6908897	−0.8744013
C	0.0000000	−3.6790168	−0.7057536
C	1.2077102	−3.7672005	−1.4166929
H	2.1507092	−3.6908897	−0.8744013
C	1.2174871	−3.9289374	−2.7979037
C	0.0000000	−4.0089268	−3.4764978
C	−1.2174871	−3.9289374	−2.7979037
C	−1.2077102	−3.7672005	−1.4166929
H	−2.1507092	−3.6908897	−0.8744013
C	−3.7283012	0.0000000	−0.6454081
H	−4.2794062	−0.8952007	−0.9696265
H	−4.2794062	0.8952007	−0.9696265
H	−2.7548060	0.0000000	−1.1546248
C	3.7283012	0.0000000	−0.6454081
H	4.2794062	0.8952007	−0.9696265
H	4.2794062	−0.8952007	−0.9696265
H	2.7548060	0.0000000	−1.1546248
H	2.1481320	3.9874205	−3.3587932
H	−2.1481320	3.9874205	−3.3587932
H	−2.1481320	−3.9874205	−3.3587932
H	2.1481320	−3.9874205	−3.3587932
N	0.0000000	−4.1947246	−4.9380991
O	1.0932607	−4.2748476	−5.5148921
O	−1.0932607	−4.2748476	−5.5148921
N	0.0000000	4.1947246	−4.9380991
O	1.0932607	4.2748476	−5.5148921
O	−1.0932607	4.2748476	−5.5148921

CH_3CN@8

N	−1.4756383	−1.9072597	−0.9214717
H	−0.9115953	−1.2185486	−0.4310436
C	−2.8177507	−1.7521384	−1.2070999
C	−3.2381059	−2.9238910	−1.8135198
H	−4.2418349	−3.1334108	−2.1670973
C	−2.1167953	−3.8020297	−1.8835008

H	−2.1097276	−4.8002070	−2.3071049
C	−1.0324593	−3.1544434	−1.3123243
C	0.3754957	−3.6309864	−1.0154897
N	1.7836119	−1.5179668	−1.0273316
H	1.4583620	−1.2018497	−0.1179401
C	1.4166814	−2.7110164	−1.6231603
C	2.2042125	−2.8443605	−2.7554899
H	2.1655192	−3.6673366	−3.4607337
C	3.0808334	−1.7230785	−2.8190337
H	3.8281436	−1.5337422	−3.5820636
C	2.8137624	−0.9146935	−1.7279354
C	3.4555151	0.3766996	−1.2555083
N	1.3312115	1.7392431	−1.3468038
H	0.7787015	1.0308829	−0.8745036
C	2.6566784	1.5815582	−1.7013488
C	3.0386722	2.7434922	−2.3508136
H	4.0230986	2.9523281	−2.7550751
C	1.9113281	3.6156740	−2.3797047
H	1.8793398	4.6069480	−2.8181105
C	0.8600725	2.9749814	−1.7426520
C	−0.5175259	3.4740359	−1.3525282
N	−1.8939642	1.3755398	−0.9712159
H	−1.4479165	1.1508897	−0.0865011
C	−1.6144317	2.4980010	−1.7302439
C	−2.5544167	2.5287092	−2.7477219
H	−2.6170102	3.2771536	−3.5301948
C	−3.4323688	1.4203051	−2.5720179
H	−4.2794727	1.1655999	−3.1996373
C	−3.0142116	0.7218560	−1.4527367
C	−3.5754138	−0.5155143	−0.7754015
C	0.5624022	−5.0473626	−1.5866943
H	−0.1516910	−5.7413588	−1.1231539
H	1.5802497	−5.4039141	−1.3815540
H	0.4003093	−5.0659636	−2.6724502
C	4.8890135	0.4757730	−1.8286186
H	4.8586856	0.5306610	−2.9244935
H	5.4749368	−0.4041692	−1.5361306
H	5.3876590	1.3757891	−1.4468117
C	−0.7846568	4.8212654	−2.0469329
H	−0.0397732	5.5653859	−1.7347316
H	−1.7813862	5.1926121	−1.7754532
H	−0.7331967	4.7238440	−3.1391540

C	−5.0682152	−0.6715016	−1.1487936
H	−5.6254586	0.2268239	−0.8556930
H	−5.4961503	−1.5410746	−0.6343287
H	−5.1787956	−0.8164327	−2.2313391
C	3.5769747	0.3543244	0.2829615
C	3.2967421	1.4908403	1.0570108
H	3.0097704	2.4178559	0.5608084
C	3.3584417	1.4419530	2.4471999
C	3.7266384	0.2458869	3.0647293
C	4.0680037	−0.8866729	2.3226192
C	3.9828651	−0.8238960	0.9358874
H	4.2076211	−1.7105674	0.3415063
C	−3.5025599	−0.3558801	0.7570576
C	−3.7331688	0.8975261	1.3520086
H	−3.9850968	1.7496333	0.7195571
C	−3.6054237	1.0757231	2.7252706
C	−3.2566308	−0.0230918	3.5143073
C	−3.0846069	−1.2934331	2.9632682
C	−3.2121554	−1.4507335	1.5863021
H	−3.0573316	−2.4321557	1.1377921
C	0.0399365	0.2870307	2.3194604
N	0.0375763	0.0227557	1.1873979
C	0.0375557	0.6245224	3.7271166
H	1.0402004	0.9442024	4.0494431
H	−0.6757526	1.4402306	3.9101958
H	−0.2690049	−0.2478659	4.3217825
C	−0.5354561	3.7065041	0.1851190
H	0.2100454	4.4679707	0.4524876
H	−1.5282096	4.0454956	0.5145555
H	−0.2781896	2.7917492	0.7392737
C	0.5651997	−3.6978743	0.5251452
H	−0.1528788	−4.4103619	0.9542707
H	1.5848367	−4.0235869	0.7752921
H	0.3866045	−2.7263127	1.0092198
H	3.1249442	2.3131882	3.0570436
H	4.3729569	−1.7957704	2.8380680
H	−2.8330950	−2.1306174	3.6116601
H	−3.7556784	2.0470749	3.1925510
N	−3.0095159	0.1768624	4.9547738
O	−2.6627910	−0.8052946	5.6228356
O	−3.1258105	1.3223656	5.4068080
N	3.7310076	0.1680422	4.5356594

O	3.0985361	1.0405779	5.1542053
O	4.3423660	−0.7600026	5.0687418

Cl^{-}@**9**

Cl	0.0000548	−0.0007269	−0.3647138
N	2.2988349	−0.0666121	1.8714544
H	1.5913588	−0.0581191	1.1146547
C	2.9502998	1.0539425	2.3390145
C	3.9400954	0.6111588	3.2076920
H	4.6275087	1.2396451	3.7644562
C	3.8866075	−0.8137801	3.2368887
H	4.5327900	−1.4722785	3.8084195
C	2.8645547	−1.2117838	2.3857286
C	2.4573287	−2.5889074	1.8803584
N	−0.0656530	−2.2991651	1.8713158
H	−0.0573853	−1.5924047	1.1138147
C	1.0550471	−2.9504021	2.3388356
C	0.6125937	−3.9387587	3.2093162
H	1.2412660	−4.6259484	3.7661511
C	−0.8123068	−3.8844824	3.2398294
H	−1.4705565	−4.5293090	3.8131775
C	−1.2106572	−2.8635978	2.3873972
C	−2.5879564	−2.4569533	1.8819511
N	−2.2989430	0.0661012	1.8706648
H	−1.5917969	0.0573906	1.1135895
C	−2.9501411	−1.0543310	2.3387884
C	−3.9394524	−0.6113626	3.2079340
H	−4.6268139	−1.2397341	3.7649001
C	−3.8857791	0.8135944	3.2369822
H	−4.5313155	1.4722087	3.8091117
C	−2.8642488	1.2113991	2.3850864
C	−2.4568525	2.5884128	1.8795356
N	0.0659943	2.2983373	1.8713234
H	0.0577409	1.5913782	1.1140864
C	−1.0546933	2.9500283	2.3381730
C	−0.6123212	3.9392677	3.2076758
H	−1.2410433	4.6269676	3.7638320
C	0.8125644	3.8849261	3.2383966
H	1.4707933	4.5302343	3.8112247
C	1.2109386	2.8632500	2.3869263
C	2.5882704	2.4564457	1.8817793

C	3.4458265	−3.6413638	2.4265392
H	4.4644974	−3.4092470	2.0904000
H	3.1701134	−4.6360151	2.0528630
H	3.4359957	−3.6599678	3.5254323
C	−3.6400006	−3.4451886	2.4294306
H	−3.6584879	−3.4339986	3.5283141
H	−3.4075002	−4.4641723	2.0945031
H	−4.6348087	−3.1703222	2.0555252
C	−3.4455226	3.6409758	2.4251995
H	−4.4640928	3.4087381	2.0888301
H	−3.1697367	4.6355535	2.0513742
H	−3.4359865	3.6598326	3.5240940
C	3.6402883	3.4446957	2.4293125
H	3.4079889	4.4636593	2.0942162
H	4.6351384	3.1696988	2.0556387
H	3.6585540	3.4336645	3.5281984
C	2.5716199	−2.5698941	0.3341563
C	1.5960915	−3.0980873	−0.5101067
H	0.7321641	−3.6080004	−0.0901297
C	1.6417991	−2.9114986	−1.8950584
C	2.7032412	−2.2046166	−2.4611396
C	3.7541123	−1.7654599	−1.6424491
C	3.6752359	−1.9380795	−0.2658233
H	4.4601600	−1.5242997	0.3717388
C	−2.5697090	−2.5731854	0.3359267
C	−3.1003073	−1.5996962	−0.5091913
H	−3.6098181	−0.7351690	−0.0898585
C	−2.9169203	−1.6483546	−1.8945141
C	−2.2100977	−2.7102565	−2.4598999
C	−1.7672854	−3.7584874	−1.6398085
C	−1.9370565	−3.6768740	−0.2630134
H	−1.5203945	−4.4595774	0.3754005
C	−2.5705645	2.5692841	0.3333415
C	−3.6733492	1.9364471	−0.2670478
H	−4.4578925	1.5215151	0.3702364
C	−3.7518411	1.7643585	−1.6437344
C	−2.7015328	2.2053308	−2.4621394
C	−1.6407963	2.9129670	−1.8955815
C	−1.5953743	3.0988206	−0.5104919
H	−0.7320084	3.6094803	−0.0901578
C	2.5700540	2.5724760	0.3356993
C	1.9397844	3.6776017	−0.2631205

H	1.5254939	4.4615421	0.3753138
C	1.7693605	3.7592778	−1.6398311
C	2.2090519	2.7097041	−2.4599190
C	2.9135360	1.6461760	−1.8947261
C	3.0979439	1.5976183	−0.5095394
H	3.6060070	0.7321632	−0.0904251
H	3.2617634	0.8097360	−2.4974647
H	1.2443558	4.6016484	−2.0930087
H	−0.8039569	3.2625140	−2.4970029
H	−4.5921860	1.2370843	−2.0980431
H	4.5950681	−1.2388550	−2.0963899
H	0.8046508	−3.2597965	−2.4967579
H	−1.2405928	−4.5997345	−2.0930935
H	−3.2678740	−0.8131447	−2.4973484
O	2.7899069	−1.8663553	−3.7965261
O	1.8739385	2.7999999	−3.7958692
O	−2.7880982	1.8680752	−3.7977897
O	−1.8756196	−2.8000742	−3.7960171
C	−2.1742072	−1.6619809	−4.6024572
H	−1.7226316	−0.7443941	−4.1904248
H	−1.7504746	−1.8751721	−5.5918748
H	−3.2617950	−1.5011510	−4.6992442
C	1.6479369	−2.1584043	−4.5999361
H	0.7335389	−1.7048408	−4.1830338
H	1.8590969	−1.7321754	−5.5887188
H	1.4828264	−3.2450631	−4.6997209
C	2.1688116	1.6607328	−4.6020024
H	1.7146048	0.7447297	−4.1893705
H	1.7452999	1.8747931	−5.5913280
H	3.2558552	1.4965170	−4.6991373
C	−1.6469172	2.1626596	−4.6013470
H	−0.7316741	1.7101711	−4.1852052
H	−1.8577501	1.7370017	−5.5904490
H	−1.4835768	3.2496788	−4.7001687

CH_3CN@9

N	−2.4635690	1.8157509	0.1866052
H	−1.6512496	1.2057026	0.1220875
C	−3.3390851	2.0646877	−0.8519172
C	−4.1816248	3.0799446	−0.4254463
H	−5.0011668	3.5070069	−0.9928465

C	−3.7798410	3.4595645	0.8897130
H	−4.2322647	4.2312140	1.5029714
C	−2.6989992	2.6691043	1.2453136
C	−1.7715609	2.7114595	2.4475714
N	−1.2481234	0.2579818	2.7804741
H	−0.6342138	0.2188531	1.9695397
C	−1.8457014	1.4159813	3.2343898
C	−2.4086850	1.1152730	4.4649899
H	−2.9732057	1.7986907	5.0902201
C	−2.1096277	−0.2484545	4.7615357
H	−2.3953277	−0.7897675	5.6571776
C	−1.3693710	−0.7575106	3.7063064
C	−0.6104222	−2.0626609	3.5313674
N	−1.3203606	−2.5158429	1.1317814
H	−0.9354510	−1.6463405	0.7698365
C	−1.2217825	−2.9284047	2.4466027
C	−1.7152099	−4.2234588	2.4923153
H	−1.8006326	−4.8408205	3.3796676
C	−2.0935700	−4.5965968	1.1691860
H	−2.5086208	−5.5510576	0.8642675
C	−1.8253857	−3.5235754	0.3350843
C	−1.8581003	−3.3961188	−1.1774178
N	−2.4673628	−0.9574470	−1.4978530
H	−1.5402934	−0.6273774	−1.2407670
C	−2.8038954	−2.2912528	−1.6116321
C	−4.0630588	−2.3333344	−2.1896372
H	−4.6340027	−3.2300217	−2.4054384
C	−4.4701605	−0.9909961	−2.4514512
H	−5.4019615	−0.6800657	−2.9114768
C	−3.4538976	−0.1502178	−2.0275792
C	−3.2400964	1.3465839	−2.1840112
C	−2.1788432	3.8847450	3.3645217
H	−2.1058237	4.8322176	2.8155165
H	−1.5082404	3.9263930	4.2317685
H	−3.2111123	3.7659750	3.7211506
C	−0.6358269	−2.8476476	4.8610478
H	−1.6660098	−3.1059680	5.1404470
H	−0.1967250	−2.2456649	5.6657210
H	−0.0532500	−3.7720707	4.7609570
C	−2.3286139	−4.7328029	−1.7912699
H	−1.6352934	−5.5367482	−1.5138242
H	−2.3515177	−4.6496885	−2.8849570

H	−3.3331860	−4.9975368	−1.4347327
C	−4.3106959	1.9161506	−3.1395067
H	−4.2537671	1.4131941	−4.1124957
H	−4.1429884	2.9901736	−3.2898975
H	−5.3186644	1.7701775	−2.7282683
C	−0.3267058	2.9682868	1.9492609
C	0.7921817	2.4182558	2.5794868
H	0.6651972	1.8100005	3.4736519
C	2.0823263	2.5808261	2.0648098
C	2.2712833	3.3338782	0.9029236
C	1.1736015	3.9739489	0.3092680
C	−0.1020270	3.7861774	0.8290015
H	−0.9555276	4.2694106	0.3495586
C	0.8627681	−1.6939623	3.2133302
C	1.5822788	−2.2479931	2.1560064
H	1.1180559	−3.0033176	1.5270534
C	2.8876923	−1.8366262	1.8522957
C	3.5041079	−0.8608513	2.6395999
C	2.8174703	−0.3332730	3.7450451
C	1.5168935	−0.7373230	4.0124930
H	0.9776172	−0.2855780	4.8479839
C	−0.4193776	−3.1212923	−1.6831328
C	0.6852440	−3.6554205	−0.9984547
H	0.5088199	−4.2895847	−0.1277168
C	1.9898848	−3.3838028	−1.3952878
C	2.2268022	−2.5647116	−2.5094417
C	1.1416790	−2.0805424	−3.2470320
C	−0.1627516	−2.3680752	−2.8310399
H	−0.9946177	−1.9634305	−3.4062696
C	−1.8489938	1.5550585	−2.8348512
C	−1.4646226	0.7452937	−3.9188247
H	−2.1697969	0.0051134	−4.3028084
C	−0.2002700	0.8412908	−4.4853639
C	0.7337941	1.7477070	−3.9616263
C	0.3528094	2.6058061	−2.9276331
C	−0.9343507	2.5067901	−2.3853746
H	−1.2070217	3.1700985	−1.5674624
H	1.0489081	3.3292810	−2.5084810
H	0.0920621	0.2063907	−5.3223738
H	1.2927963	−1.4564008	−4.1246606
H	2.8411736	−3.7946823	−0.8513484
H	1.3392641	4.6050070	−0.5643399

H	2.9149906	2.0865227	2.5601969
H	3.3173197	0.4079591	4.3698398
H	3.3935399	−2.2808632	0.9967110
O	3.4794823	3.4700230	0.2527857
O	2.0035262	1.6856056	−4.4955622
O	3.5427586	−2.2682014	−2.7768566
O	4.7582937	−0.3421195	2.4097411
C	5.5377151	−0.9446301	1.3777529
H	5.0746463	−0.8165489	0.3834105
H	6.5049742	−0.4299573	1.3917109
H	5.6906464	−2.0206408	1.5633671
C	4.6372746	2.9512385	0.9176101
H	4.5679354	1.8625386	1.0816936
H	5.4835646	3.1729105	0.2579568
H	4.7903224	3.4439606	1.8917307
C	2.9239029	2.7130645	−4.1172285
H	3.1496490	2.6894607	−3.0376190
H	3.8408370	2.5162186	−4.6838375
H	2.5362356	3.7110028	−4.3791641
C	3.8208271	−1.5559507	−3.9861248
H	3.3543760	−0.5557103	−3.9978910
H	4.9108088	−1.4515450	−4.0262136
H	3.4737172	−2.1187118	−4.8685721
N	0.0915802	0.0393816	−0.0193727
C	1.1317265	0.2208683	−0.5007847
C	2.4214692	0.4307353	−1.1134146
H	2.8394771	1.3922997	−0.7850016
H	3.0917608	−0.3944074	−0.8384794
H	2.2964334	0.4363680	−2.2043377

Cl^{-}@**10**

Cl	−0.0002246	−0.0003562	−1.1013664
N	0.7987707	2.1562389	1.1034507
H	0.5389905	1.4886447	0.3540493
C	−0.0516853	3.1055648	1.6240205
C	0.7006860	3.9052549	2.4732138
H	0.3328023	4.7505752	3.0458016
C	2.0429108	3.4255410	2.4378928
H	2.8837468	3.8298652	2.9915711
C	2.0821452	2.3435620	1.5669823
C	3.2433899	1.4781774	1.1140059

N	2.1556858	−0.7990696	1.1041162
H	1.4887912	−0.5395660	0.3540212
C	3.1054106	0.0513812	1.6239831
C	3.9058237	−0.7010060	2.4724852
H	4.7514017	−0.3330664	3.0446545
C	3.4261212	−2.0432405	2.4374760
H	3.8307714	−2.8840559	2.9909456
C	2.3434719	−2.0824499	1.5674037
C	1.4779594	−3.2437407	1.1148735
N	−0.7994378	−2.1563596	1.1032482
H	−0.5396119	−1.4888891	0.3537129
C	0.0510635	−3.1052585	1.6244880
C	−0.7013287	−3.9045130	2.4740920
H	−0.3334432	−4.7494751	3.0472076
C	−2.0435744	−3.4248878	2.4384383
H	−2.8844401	−3.8289696	2.9922447
C	−2.0828053	−2.3434568	1.5668498
C	−3.2439069	−1.4780588	1.1135308
N	−2.1571255	0.7994682	1.1024553
H	−1.4892340	0.5395717	0.3533687
C	−3.1056380	−0.0513864	1.6238148
C	−3.9042593	0.7004512	2.4744925
H	−4.7489644	0.3322525	3.0477903
C	−3.4245770	2.0427047	2.4394074
H	−3.8283504	2.8832514	2.9939247
C	−2.3437240	2.0824863	1.5670921
C	−1.4785021	3.2438373	1.1141036
C	4.5556669	2.0861137	1.6558138
H	4.6775888	3.1075853	1.2728714
H	5.4129820	1.4839196	1.3297433
H	4.5474842	2.1175281	2.7541155
C	2.0855137	−4.5559627	1.6571986
H	2.1169781	−4.5473322	2.7554921
H	3.1069392	−4.6783691	1.2742837
H	1.4830075	−5.4131861	1.3314781
C	−4.5563596	−2.0857779	1.6552188
H	−4.6784889	−3.1072055	1.2722200
H	−5.4134934	−1.4833024	1.3291933
H	−4.5482423	−2.1173112	2.7535180
C	−2.0863232	4.5560354	1.6562114
H	−3.1078208	4.6780733	1.2733783
H	−1.4840685	5.4133759	1.3303252

H	−2.1176807	4.5476303	2.7545126
C	3.3562893	1.4056175	−0.4308725
C	4.1579236	0.4035433	−0.9995963
H	4.7001673	−0.2805028	−0.3424217
C	4.2322883	0.2261358	−2.3791286
C	3.4801014	1.0610216	−3.2074827
C	2.7089607	2.0909952	−2.6795428
C	2.6699479	2.2646794	−1.2931570
H	2.0384795	3.0523942	−0.8872900
C	1.4057527	−3.3569405	−0.4299737
C	0.4032051	−4.1577778	−0.9989633
H	−0.2815075	−4.6993871	−0.3419712
C	0.2262995	−4.2322119	−2.3785534
C	1.0622675	−3.4809901	−3.2066851
C	2.0928153	−2.7107876	−2.6784590
C	2.2659578	−2.6716870	−1.2920065
H	3.0541099	−2.0409470	−0.8858025
C	−3.3566180	−1.4055783	−0.4312644
C	−2.6709604	−2.2654151	−1.2933120
H	−2.0401966	−3.0535462	−0.8871538
C	−2.7096506	−2.0918937	−2.6797259
C	−3.4798584	−1.0613357	−3.2079013
C	−4.2315195	−0.2257523	−2.3797728
C	−4.1575207	−0.4030605	−1.0002076
H	−4.6994735	0.2813842	−0.3432078
C	−1.4059590	3.3569923	−0.4307399
C	−2.2652391	2.6711214	−1.2931701
H	−3.0532559	2.0399480	−0.8874397
C	−2.0913541	2.7101597	−2.6795389
C	−1.0610166	3.4809242	−3.2072987
C	−0.2259737	4.2327724	−2.3787985
C	−0.4036119	4.1584084	−0.9992972
H	0.2804932	4.7004494	−0.3420238
H	0.5728494	4.8398080	−2.8040441
H	−2.7223749	2.1054410	−3.3302530
H	−4.8381687	0.5731854	−2.8053628
H	−2.1051620	−2.7233810	−3.3301942
H	2.1039610	2.7218773	−3.3301317
H	4.8395436	−0.5724491	−2.8045146
H	2.7245704	−2.1066049	−3.3289578
H	−0.5726336	−4.8388334	−2.8041801
Br	−3.4765449	−0.7396933	−5.0993098

Br	0.7412807	−3.4783169	−5.0982027
Br	−0.7389170	3.4781143	−5.0986323
Br	3.4774598	0.7392822	−5.0988744

CH_3CN@10

N	1.2890767	1.9020259	1.3056509
H	0.8865715	1.3331784	0.5634192
C	0.6922248	3.0379741	1.8153139
C	1.6207815	3.6382030	2.6497091
H	1.4713960	4.5521601	3.2139708
C	2.8083311	2.8499980	2.6163036
H	3.7212662	3.0457145	3.1678261
C	2.5883505	1.7832863	1.7584498
C	3.5040973	0.6615966	1.3109302
N	1.8893203	−1.2798925	1.3018611
H	1.3233335	−0.8734760	0.5597722
C	3.0218998	−0.6859446	1.8219019
C	3.6147740	−1.6176117	2.6581054
H	4.5220923	−1.4692395	3.2332378
C	2.8280157	−2.8057423	2.6115031
H	3.0222225	−3.7231659	3.1560981
C	1.7670203	−2.5809155	1.7479025
C	0.6513029	−3.4946791	1.2827291
N	−1.2943581	−1.8855469	1.2857716
H	−0.8910009	−1.3145621	0.5458064
C	−0.7001157	−3.0230882	1.7936183
C	−1.6289275	−3.6206739	2.6298978
H	−1.4799850	−4.5331227	3.1967381
C	−2.8151506	−2.8297270	2.5968139
H	−3.7305881	−3.0260399	3.1440852
C	−2.5916528	−1.7620868	1.7412315
C	−3.5054529	−0.6453852	1.2776074
N	−1.8968129	1.3029570	1.2879004
H	−1.3235676	0.9028625	0.5476920
C	−3.0365175	0.7068376	1.7891911
C	−3.6398900	1.6345469	2.6225064
H	−4.5558794	1.4846020	3.1833461
C	−2.8496615	2.8213111	2.5960099
H	−3.0486962	3.7348343	3.1454616
C	−1.7774055	2.6001165	1.7455028
C	−0.6546642	3.5147681	1.2973203

C	4.9258628	0.9339416	1.8491996
H	5.2901096	1.8956948	1.4676295
H	5.6134108	0.1439393	1.5229049
H	4.9262131	0.9642932	2.9469601
C	0.9252742	−4.9216659	1.8064291
H	0.9506828	−4.9341330	2.9042692
H	1.8897822	−5.2790026	1.4253147
H	0.1384483	−5.6075999	1.4691174
C	−4.9347674	−0.9200031	1.7941325
H	−5.2897217	−1.8846495	1.4111204
H	−5.6184819	−0.1329856	1.4522840
H	−4.9532412	−0.9455269	2.8919442
C	−0.9297227	4.9401365	1.8243343
H	−1.8908381	5.3005638	1.4375301
H	−0.1391100	5.6252102	1.4938620
H	−0.9629468	4.9491249	2.9220349
C	3.5908245	0.5567343	−0.2340556
C	4.1172868	−0.6157804	−0.8005901
H	4.4797877	−1.4072746	−0.1406877
C	4.1479127	−0.8106489	−2.1791765
C	3.6327667	0.1851906	−3.0120097
C	3.1500979	1.3813175	−2.4869670
C	3.1498020	1.5621905	−1.0995190
H	2.7628844	2.4952294	−0.6951277
C	0.5602517	−3.5650401	−0.2639899
C	−0.5999788	−4.1009706	−0.8459474
H	−1.3933888	−4.4773367	−0.1963202
C	−0.7774305	−4.1305344	−2.2268846
C	0.2216453	−3.6014109	−3.0472913
C	1.4048927	−3.1045182	−2.5061335
C	1.5706005	−3.1091225	−1.1167061
H	2.4963814	−2.7162317	−0.7011174
C	−3.5664901	−0.5613620	−0.2693070
C	−3.1430548	−1.5973118	−1.1083120
H	−2.7829342	−2.5301885	−0.6786228
C	−3.1283781	−1.4474049	−2.4987338
C	−3.5712338	−0.2497356	−3.0561041
C	−4.0713634	0.7746930	−2.2493416
C	−4.0599923	0.6092833	−0.8662894
H	−4.4152146	1.4203709	−0.2266780
C	−0.5525482	3.5905799	−0.2479586
C	−1.5761698	3.1715056	−1.1033806

H	−2.5134718	2.8066334	−0.6880485
C	−1.4076651	3.1669828	−2.4918039
C	−0.2024484	3.6132473	−3.0293461
C	0.8108597	4.1089935	−2.2062622
C	0.6254457	4.0903248	−0.8258031
H	1.4277431	4.4420789	−0.1731472
C	−0.0382774	−0.0061674	−1.9927906
N	−0.0140045	0.0222907	−0.8313039
C	−0.0725160	−0.0436392	−3.4318896
H	0.9503343	−0.0686848	−3.8326698
H	−0.6095189	−0.9399956	−3.7688797
H	−0.5878204	0.8459722	−3.8178449
H	1.7396359	4.4820784	−2.6364964
H	−2.1994046	2.7953963	−3.1422542
H	−4.4470079	1.6951862	−2.6948613
H	−2.7530354	−2.2484553	−3.1356718
H	−1.6884889	−4.5412121	−2.6607346
H	2.1817265	−2.6966690	−3.1532735
H	4.5432532	−1.7341325	−2.6008256
H	2.7540946	2.1555072	−3.1444499
Br	0.0965309	3.4969640	−4.9178655
Br	3.5384207	−0.1326096	−4.9001245
Br	−0.0718900	−3.5173464	−4.9389281
Br	−3.4521567	0.0216222	−4.9477255

Cl^{-}@**11**

Cl	0.0000225	−0.0002864	−1.1573139
N	−0.6037731	−2.2346271	1.0331375
H	−0.4257903	−1.5534426	0.2712544
C	−1.8461550	−2.5035644	1.5621463
C	−1.6937500	−3.5646860	2.4441429
H	−2.4819740	−4.0238343	3.0317986
C	−0.3205074	−3.9457015	2.4243034
H	0.1330085	−4.7446439	3.0017533
C	0.3362175	−3.1094726	1.5304572
C	1.7833369	−3.0919541	1.0754313
N	2.2343665	−0.6036855	1.0334654
H	1.5527569	−0.4263533	0.2718299
C	2.5030795	−1.8456955	1.5635286
C	3.5640823	−1.6926733	2.4455689
H	4.0229307	−2.4804351	3.0340858

C	3.9454995	−0.3195696	2.4243688
H	4.7446021	0.1343115	3.0013048
C	3.1094143	0.3365065	1.5299028
C	3.0919927	1.7832895	1.0737927
N	0.6038035	2.2344936	1.0330296
H	0.4259741	1.5535740	0.2708898
C	1.8460715	2.5032599	1.5623865
C	1.6934668	3.5640177	2.4447844
H	2.4815544	4.0228237	3.0328951
C	0.3202653	3.9451560	2.4246546
H	−0.1334098	4.7438539	3.0023207
C	−0.3362391	3.1093236	1.5302739
C	−1.7832525	3.0918588	1.0749368
N	−2.2345159	0.6036650	1.0328526
H	−1.5534474	0.4264177	0.2707149
C	−2.5029726	1.8456315	1.5631305
C	−3.5635603	1.6925610	2.4456597
H	−4.0222538	2.4803368	3.0342803
C	−3.9448152	0.3193995	2.4247566
H	−4.7435317	−0.1345744	3.0021531
C	−3.1091497	−0.3366112	1.5298464
C	−3.0919587	−1.7833634	1.0736251
C	2.4963738	−4.3389788	1.6467823
H	1.9993154	−5.2479539	1.2842289
H	3.5430097	−4.3542029	1.3176795
H	2.4731988	−4.3354600	2.7454903
C	4.3393976	2.4965096	1.6440232
H	4.3365857	2.4739505	2.7427436
H	5.2481022	1.9991541	1.2812010
H	4.3544801	3.5429701	1.3143611
C	−2.4963853	4.3389645	1.6460235
H	−1.9992797	5.2478989	1.2834323
H	−3.5429885	4.3541134	1.3168156
H	−2.4733536	4.3356051	2.7447347
C	−4.3395065	−2.4964967	1.6437018
H	−5.2481069	−1.9988707	1.2809956
H	−4.3547414	−3.5428681	1.3137617
H	−4.3367011	−2.4742094	2.7424272
C	1.9039566	−3.1682170	−0.4668249
C	3.1097614	−2.7951417	−1.0795654
H	3.9290951	−2.4349266	−0.4534134
C	3.2599640	−2.8504740	−2.4645715

H	4.2023797	−2.5373335	−2.9183740
C	2.1999968	−3.2807798	−3.2687193
C	1.0032139	−3.6723480	−2.6680509
H	0.1569386	−3.9875039	−3.2806677
C	0.8613891	−3.6262212	−1.2792324
H	−0.0863436	−3.9131243	−0.8272584
C	3.1674197	1.9029571	−0.4685443
C	2.7944122	3.1085547	−1.0817527
H	2.4349528	3.9284279	−0.4558717
C	2.8489202	3.2579408	−2.4668775
H	2.5359219	4.2002354	−2.9210355
C	3.2782971	2.1973379	−3.2707010
C	3.6698452	1.0007937	−2.6695823
H	3.9843449	0.1539702	−3.2817762
C	3.6245413	0.8597884	−1.2806394
H	3.9113506	−0.0878807	−0.8285010
C	−1.9035955	3.1679321	−0.4673136
C	−0.8607512	3.6252902	−1.2797092
H	0.0870899	3.9119387	−0.8278079
C	−1.0024367	3.6712170	−2.6685558
H	−0.1558988	3.9857856	−3.2811109
C	−2.1993809	3.2802095	−3.2692587
C	−3.2596373	2.8505840	−2.4651150
H	−4.2022122	2.5379417	−2.9189346
C	−3.1095476	2.7953625	−1.0800928
H	−3.9291522	2.4357350	−0.4539538
C	−3.1674991	−1.9026544	−0.4686954
C	−3.6259212	−0.8595768	−1.2802342
H	−3.9135254	0.0875714	−0.8274726
C	−3.6715672	−1.0000879	−2.6691876
H	−3.9870584	−0.1533862	−3.2810488
C	−3.2791628	−2.1960924	−3.2708956
C	−2.8485481	−3.2565924	−2.4676356
H	−2.5349782	−4.1984831	−2.9222339
C	−2.7936284	−3.1076569	−1.0824608
H	−2.4331982	−3.9274283	−0.4570205
H	−2.3021197	3.2942066	−4.3557929
H	3.2916379	2.2995203	−4.3572954
H	2.3028378	−3.2949492	−4.3552408
H	−3.2928933	−2.2978742	−4.3575254

CH_3CN@11

N	−1.9864979	−1.1176632	1.2757509
H	−1.4029334	−0.7733468	0.5169022
C	−1.9632925	−2.4162267	1.7438074
C	−3.0238907	−2.5369247	2.6283467
H	−3.2846111	−3.4263311	3.1914047
C	−3.7082899	−1.2860535	2.6657204
H	−4.5892775	−1.0526124	3.2535022
C	−3.0539922	−0.4210044	1.8037716
C	−3.4100179	0.9682274	1.3040666
N	−1.0973067	2.0086435	1.2744819
H	−0.7587220	1.4067433	0.5272288
C	−2.3917126	1.9974212	1.7537392
C	−2.5044792	3.0783802	2.6145700
H	−3.3888663	3.3527818	3.1790105
C	−1.2526862	3.7620820	2.6260140
H	−1.0136506	4.6560698	3.1915979
C	−0.3953048	3.0870405	1.7722781
C	0.9893248	3.4312849	1.2509980
N	2.0281410	1.1192983	1.2397852
H	1.4206101	0.7762071	0.4987276
C	2.0211826	2.4179883	1.7065572
C	3.1092832	2.5387190	2.5573533
H	3.3885323	3.4284339	3.1110238
C	3.7931147	1.2868916	2.5744786
H	4.6919500	1.0530703	3.1345640
C	3.1107788	0.4217747	1.7341592
C	3.4515328	−0.9660982	1.2187267
N	1.1378550	−2.0025896	1.2509392
H	0.7771223	−1.3852300	0.5269737
C	2.4463407	−1.9984177	1.6903644
C	2.5862093	−3.0948133	2.5273513
H	3.4877223	−3.3781762	3.0593724
C	1.3359994	−3.7805675	2.5641384
H	1.1150233	−4.6849266	3.1204495
C	0.4524507	−3.0911252	1.7492952
C	−0.9451289	−3.4302010	1.2609533
C	−4.7974207	1.3660281	1.8559069
H	−5.5544478	0.6429301	1.5285285
H	−5.0751650	2.3596785	1.4832780
H	−4.7869847	1.3891456	2.9539273

C	1.3964620	4.8274676	1.7721783
H	1.4314849	4.8378444	2.8699438
H	0.6716623	5.5801072	1.4383437
H	2.3867273	5.0955480	1.3837044
C	4.8541800	−1.3671362	1.7268768
H	5.6015838	−0.6425810	1.3810652
H	5.1208048	−2.3587992	1.3409972
H	4.8763314	−1.3958727	2.8246591
C	−1.3427769	−4.8267493	1.7890849
H	−0.6286032	−5.5804451	1.4352082
H	−2.3431109	−5.0914305	1.4249997
H	−1.3495646	−4.8396510	2.8873563
C	−3.5262061	0.9116208	−0.2422662
C	−3.0656947	1.9392038	−1.0732814
H	−2.5975645	2.8179944	−0.6338912
C	−3.1829178	1.8458601	−2.4639481
C	−3.7840162	0.7295703	−3.0488871
C	−4.2756634	−0.2906453	−2.2269517
C	−4.1448810	−0.1977505	−0.8417229
H	−4.5183351	−1.0010044	−0.2027215
C	0.9144350	3.5153591	−0.2968216
C	1.9338777	3.0409860	−1.1301126
H	2.8231866	2.5924770	−0.6914503
C	1.8170518	3.1151105	−2.5218087
C	0.6856535	3.6868314	−3.1065887
C	−0.3227309	4.1996115	−2.2834129
C	−0.2067314	4.1117033	−0.8965565
H	−1.0021089	4.4993784	−0.2562339
C	3.5164548	−0.8999146	−0.3307296
C	4.1206806	0.2101443	−0.9428406
H	4.5294487	1.0018241	−0.3111312
C	4.1874288	0.3214915	−2.3313059
C	3.6413780	−0.6783108	−3.1435092
C	3.0614433	−1.7990497	−2.5467144
C	3.0122124	−1.9132895	−1.1538869
H	2.5575286	−2.7947367	−0.7061168
C	−0.9106479	−3.5136747	−0.2883714
C	0.1921317	−4.1136481	−0.9172718
H	1.0053976	−4.4987766	−0.2983017
C	0.2658128	−4.2119919	−2.3066394
C	−0.7665684	−3.7035801	−3.1025941
C	−1.8772297	−3.1215691	−2.4883133

C	−1.9527553	−3.0403260	−1.0943811
H	−2.8283962	−2.5881415	−0.6323460
H	3.6808217	−0.5885501	−4.2303900
H	0.5937011	3.7454636	−4.1924408
H	−3.8818777	0.6584947	−4.1337016
H	−0.7098704	−3.7738622	−4.1903766
C	−0.0065495	−0.0159053	−1.9742314
N	0.0241156	0.0123051	−0.8153023
C	−0.0484956	−0.0500701	−3.4152124
H	−1.0536695	0.2472631	−3.7454247
H	0.7002801	0.6495088	−3.8100057
H	0.1700270	−1.0719667	−3.7526707
H	−2.6937570	−2.7208811	−3.0911181
H	1.1331897	−4.6864715	−2.7687421
H	2.6374712	−2.5945566	−3.1619324
H	4.6639160	1.1946600	−2.7802437
H	2.6192009	2.7173684	−3.1457867
H	−1.2047765	4.6687902	−2.7226027
H	−2.7956177	2.6534300	−3.0872626
H	−4.7634595	−1.1626112	−2.6659091

Cl^-@**12**

Cl	−0.0000932	−0.0002083	−0.9192682
N	0.8249761	2.1568564	1.2802720
H	0.5625534	1.4953800	0.5266504
C	−0.0184208	3.1073946	1.8107555
C	0.7347857	3.8842753	2.6796445
H	0.3718732	4.7230951	3.2643654
C	2.0698572	3.3878929	2.6517047
H	2.9093772	3.7731704	3.2203082
C	2.1037779	2.3187081	1.7659320
C	3.2597118	1.4477547	1.3149608
N	2.1569774	−0.8250773	1.2799321
H	1.4945248	−0.5637929	0.5267693
C	3.1091721	0.0188153	1.8066116
C	3.8877588	−0.7330809	2.6750571
H	4.7279786	−0.3694048	3.2572852
C	3.3906813	−2.0679579	2.6508155
H	3.7768934	−2.9065095	3.2202043
C	2.3193765	−2.1031030	1.7676497
C	1.4474271	−3.2598474	1.3207804

N	−0.8250732	−2.1566838	1.2808325
H	−0.5625098	−1.4949913	0.5274835
C	0.0182806	−3.1072381	1.8113298
C	−0.7351260	−3.8845009	2.6797115
H	−0.3724019	−4.7237164	3.2639864
C	−2.0701654	−3.3880245	2.6517814
H	−2.9097333	−3.7732983	3.2203232
C	−2.1039610	−2.3186759	1.7662009
C	−3.2598590	−1.4476662	1.3152401
N	−2.1571444	0.8251611	1.2798806
H	−1.4947088	0.5637582	0.5267505
C	−3.1092541	−0.0186847	1.8067962
C	−3.8878530	0.7333763	2.6751002
H	−4.7281182	0.3698183	3.2573458
C	−3.3907576	2.0682509	2.6506303
H	−3.7769956	2.9069174	3.2198396
C	−2.3194576	2.1032329	1.7674534
C	−1.4475759	3.2599041	1.3202285
C	4.5734540	2.0392486	1.8775196
H	4.7070209	3.0651082	1.5112038
H	5.4289004	1.4345832	1.5518083
H	4.5519596	2.0546196	2.9756183
C	2.0381586	−4.5719560	1.8879735
H	2.0534524	−4.5464869	2.9859805
H	3.0639538	−4.7074398	1.5221763
H	1.4331206	−5.4282752	1.5653098
C	−4.5736101	−2.0390857	1.8777976
H	−4.7072482	−3.0649363	1.5114806
H	−5.4290462	−1.4343752	1.5521254
H	−4.5521022	−2.0544732	2.9758980
C	−2.0383030	4.5721463	1.8871183
H	−3.0641066	4.7075324	1.5213041
H	−1.4332692	5.4283862	1.5642359
H	−2.0535899	4.5469407	2.9851314
C	3.4054277	1.4232913	−0.2247154
C	4.1876917	0.4176766	−0.8164553
H	4.6438944	−0.3405492	−0.1766925
C	4.3771026	0.3660871	−2.1909407
C	3.7736350	1.3328483	−3.0186295
C	2.9884585	2.3421079	−2.4372168
C	2.8206629	2.3857632	−1.0555089
H	2.2015564	3.1679998	−0.6204368

C	1.4224561	−3.4111569	−0.2184411
C	0.4184404	−4.1983902	−0.8064170
H	−0.3382372	−4.6539326	−0.1643350
C	0.3665717	−4.3936785	−2.1800415
C	1.3312327	−3.7910257	−3.0108101
C	2.3385273	−3.0005249	−2.4332648
C	2.3825721	−2.8268877	−1.0522500
H	3.1634102	−2.2039327	−0.6202060
C	−3.4054498	−1.4230918	−0.2244759
C	−2.8198751	−2.3848989	−1.0554469
H	−2.2005659	−3.1670177	−0.6204754
C	−2.9870726	−2.3407053	−2.4372065
C	−3.7724497	−1.3315577	−3.0185150
C	−4.3770312	−0.3656540	−2.1906284
C	−4.1882435	−0.4177908	−0.8160740
H	−4.6453329	0.3397909	−0.1761627
C	−1.4227656	3.4107736	−0.2190155
C	−2.3830355	2.8263002	−1.0525131
H	−3.1639559	2.2036736	−0.6201414
C	−2.3390402	2.9993220	−2.4336046
C	−1.3316787	3.7894527	−3.0115553
C	−0.3668718	4.3923056	−2.1811095
C	−0.4186856	4.1976204	−0.8073914
H	0.3381371	4.6532790	−0.1655650
H	0.4166774	5.0066702	−2.6252091
H	−3.0755420	2.5123002	−3.0723772
H	−4.9880000	0.4190765	−2.6373392
H	−2.4988898	−3.0782662	−3.0738409
H	2.5008599	3.0801379	−3.0737724
H	4.9876780	−0.4188750	−2.6377659
H	3.0749427	−2.5136932	−3.0722793
H	−0.4168976	−5.0083772	−2.6238108
C	3.9857573	1.2935974	−4.4307125
N	4.1915281	1.2647379	−5.5784067
C	1.2919381	−4.0094974	−4.4219167
N	1.2630038	−4.2204483	−5.5686690
C	−1.2925411	4.0073397	−4.4227581
N	−1.2638802	4.2178837	−5.5695941
C	−3.9835143	−1.2913789	−4.4307291
N	−4.1881260	−1.2615417	−5.5786036

CH_3CN@12

N	1.2955797	1.8978003	1.4819535
H	0.9007814	1.3391966	0.7284194
C	0.6978705	3.0309109	1.9992670
C	1.6189074	3.6182924	2.8500387
H	1.4656731	4.5242830	3.4257387
C	2.8042065	2.8276071	2.8197485
H	3.7126919	3.0163139	3.3808156
C	2.5893469	1.7703597	1.9500270
C	3.5065811	0.6503176	1.5050718
N	1.8904781	−1.2921190	1.4872416
H	1.3327023	−0.8931709	0.7355035
C	3.0221203	−0.6970093	2.0105885
C	3.6072050	−1.6223292	2.8583105
H	4.5117301	−1.4719595	3.4370691
C	2.8166539	−2.8075273	2.8196287
H	3.0038752	−3.7190094	3.3763224
C	1.7618955	−2.5882577	1.9479465
C	0.6445575	−3.5032264	1.4921798
N	−1.2999210	−1.8895232	1.4720330
H	−0.9023044	−1.3319921	0.7192151
C	−0.7052451	−3.0215520	1.9945958
C	−1.6301695	−3.6055471	2.8437023
H	−1.4798652	−4.5098362	3.4228729
C	−2.8146473	−2.8134274	2.8066507
H	−3.7261290	−2.9998339	3.3636970
C	−2.5948933	−1.7590170	1.9346994
C	−3.5089604	−0.6432338	1.4724163
N	−1.8965397	1.3035866	1.4668821
H	−1.3339897	0.9084835	0.7162582
C	−3.0332137	0.7074538	1.9778214
C	−3.6246996	1.6298463	2.8244152
H	−4.5338101	1.4778236	3.3955380
C	−2.8321812	2.8142541	2.7981676
H	−3.0233855	3.7237079	3.3568638
C	−1.7699640	2.5971284	1.9350460
C	−0.6486481	3.5130181	1.4895830
C	4.9281207	0.9206129	2.0478629
H	5.2942416	1.8833323	1.6708771
H	5.6159545	0.1306798	1.7221815
H	4.9233870	0.9478753	3.1454953

C	0.9125683	−4.9277644	2.0280126
H	0.9358917	−4.9290877	3.1257492
H	1.8765645	−5.2920881	1.6525608
H	0.1235780	−5.6136465	1.6959889
C	−4.9373755	−0.9129286	1.9968675
H	−5.2969198	−1.8780799	1.6197747
H	−5.6211924	−0.1251520	1.6573764
H	−4.9481033	−0.9347729	3.0946274
C	−0.9198025	4.9367699	2.0262682
H	−1.8817433	5.3015360	1.6459663
H	−0.1288777	5.6227352	1.6987925
H	−0.9492590	4.9365959	3.1238869
C	3.6060089	0.5563859	−0.0369786
C	4.1247960	−0.6180178	−0.6095010
H	4.4644267	−1.4202343	0.0489545
C	4.1802893	−0.7875274	−1.9860916
C	3.7009377	0.2292177	−2.8353402
C	3.2262464	1.4276826	−2.2764623
C	3.1974391	1.5867782	−0.8920384
H	2.8215238	2.5177677	−0.4727778
C	0.5587818	−3.5922597	−0.0511224
C	−0.6102325	−4.1138142	−0.6317595
H	−1.4120759	−4.4645419	0.0211984
C	−0.7750370	−4.1582360	−2.0091807
C	0.2397097	−3.6623232	−2.8514684
C	1.4338362	−3.1862222	−2.2849037
C	1.5894926	−3.1706526	−0.8997426
H	2.5174454	−2.7930870	−0.4751375
C	−3.5855841	−0.5654645	−0.0715691
C	−3.1925152	−1.6190003	−0.9060811
H	−2.8481315	−2.5535816	−0.4676621
C	−3.1952812	−1.4780377	−2.2924614
C	−3.6220126	−0.2728805	−2.8754828
C	−4.0924017	0.7651543	−2.0477482
C	−4.0678494	0.6117910	−0.6681223
H	−4.4035954	1.4284452	−0.0258198
C	−0.5578129	3.6071270	−0.0527559
C	−1.6045457	3.2236402	−0.8997429
H	−2.5404975	2.8700507	−0.4719751
C	−1.4563300	3.2523548	−2.2850897
C	−0.2501178	3.6949159	−2.8540050
C	0.7842767	4.1486370	−2.0125584

C	0.6232345	4.0990709	−0.6343405
H	1.4361973	4.4241173	0.0182365
C	−0.0364503	−0.0106109	−1.9072599
N	−0.0156605	0.0125458	−0.7451963
C	−0.0634343	−0.0395253	−3.3527093
H	0.9602873	−0.0657207	−3.7506086
H	−0.6015024	−0.9306184	−3.7024427
H	−0.5715219	0.8545542	−3.7389446
H	1.7101910	4.5222642	−2.4497625
H	−2.2662245	2.9182327	−2.9338405
H	−4.4586819	1.6883991	−2.4966242
H	−2.8511635	−2.2924095	−2.9304288
H	−1.6905302	−4.5575479	−2.4457126
H	2.2270276	−2.8138828	−2.9336710
H	4.5754049	−1.7077651	−2.4163909
H	2.8654693	2.2214517	−2.9308826
C	−3.5456033	−0.0810379	−4.2880557
N	−3.4496202	0.0994303	−5.4359517
C	0.0293086	−3.6090664	−4.2625607
N	−0.1734859	−3.5320867	−5.4083402
C	3.6614988	0.0199594	−4.2472245
N	3.5937216	−0.1755548	−5.3949296
C	−0.0578154	3.6540063	−4.2682378
N	0.1168409	3.5892978	−5.4193119

Cl^-@**13**

Cl	−0.0000007	−0.0000397	−0.7466242
N	0.0349966	−2.3072855	1.4576979
H	0.0331705	−1.5970422	0.7028633
C	1.1715253	−2.8766399	1.9868993
C	0.7614946	−3.8808546	2.8527400
H	1.4107355	−4.5258418	3.4354241
C	−0.6624889	−3.9176271	2.8233307
H	−1.2985434	−4.5885766	3.3907808
C	−1.0914767	−2.9356807	1.9395557
C	−2.4881143	−2.5539426	1.4899746
N	−2.3072354	−0.0351110	1.4573626
H	−1.5977130	−0.0326986	0.7018480
C	−2.8762932	−1.1718366	1.9863632
C	−3.8795771	−0.7621951	2.8534686
H	−4.5240984	−1.4116813	3.4364038

C	−3.9158580	0.6618283	2.8253001
H	−4.5861617	1.2976484	3.3937899
C	−2.9347777	1.0911982	1.9407544
C	−2.5534952	2.4880325	1.4914646
N	−0.0346801	2.3070353	1.4579109
H	−0.0328923	1.5963708	0.7034703
C	−1.1711840	2.8765911	1.9869180
C	−0.7611422	3.8813487	2.8521156
H	−1.4103594	4.5266081	3.4345139
C	0.6628161	3.9182253	2.8225558
H	1.2988506	4.5896424	3.3894682
C	1.0918024	2.9357716	1.9393544
C	2.4884441	2.5538190	1.4900422
N	2.3074210	0.0350022	1.4575098
H	1.5979224	0.0325903	0.7019725
C	2.8762954	1.1717238	1.9867538
C	3.8793240	0.7620470	2.8541256
H	4.5236006	1.4115158	3.4373256
C	3.9158294	−0.6619548	2.8256883
H	4.5860279	−1.2977919	3.3942629
C	2.9350294	−1.0913164	1.9408356
C	2.5538387	−2.4881136	1.4913280
C	−3.4898568	−3.5920965	2.0467507
H	−3.2328955	−4.5929517	1.6771269
H	−4.5083417	−3.3462109	1.7213544
H	−3.4654205	−3.6024977	3.1447657
C	−3.5914910	3.4893691	2.0493315
H	−3.6007791	3.4648307	3.1473580
H	−4.5926219	3.2320889	1.6806813
H	−3.3463385	4.5079736	1.7237638
C	3.4901200	3.5918485	2.0470885
H	3.2333868	4.5927262	1.6773784
H	4.5086769	3.3458746	1.7220239
H	3.4653461	3.6022716	3.1450937
C	3.5918708	−3.4895027	2.0489729
H	4.5929531	−3.2322313	1.6801983
H	3.3466254	−4.5080858	1.7233894
H	3.6013313	−3.4650505	3.1469993
C	−2.6325250	−2.5727529	−0.0499148
C	−3.7308684	−1.9177142	−0.6337806
H	−4.4387345	−1.3957865	0.0129803
C	−3.9179112	−1.9092128	−2.0098210

C	−2.9889954	−2.5685144	−2.8180943
C	−1.8940374	−3.2349502	−2.2716903
C	−1.7297516	−3.2390253	−0.8878157
H	−0.8686791	−3.7458281	−0.4572279
C	−2.5737294	2.6331443	−0.0483904
C	−1.9184158	3.7311864	−0.6324734
H	−1.3951243	4.4382725	0.0140128
C	−1.9118122	3.9192166	−2.0083857
C	−2.5733677	2.9916415	−2.8163271
C	−3.2399551	1.8968491	−2.2697170
C	−3.2419875	1.7314789	−0.8859679
H	−3.7493025	0.8708035	−0.4551484
C	2.6334538	2.5724411	−0.0498235
C	1.7306402	3.2378379	−0.8883668
H	0.8692536	3.7446311	−0.4583962
C	1.8953243	3.2330048	−2.2722188
C	2.9907365	2.5667221	−2.8179086
C	3.9197152	1.9083349	−2.0089529
C	3.7322567	1.9175588	−0.6330063
H	4.4401215	1.3962511	0.0142290
C	2.5737262	−2.6328761	−0.0485710
C	3.2411780	−1.7306814	−0.8861867
H	3.7484319	−0.8699866	−0.4553890
C	3.2378508	−1.8951301	−2.2700416
C	2.5709710	−2.9896726	−2.8167424
C	1.9105498	−3.9180419	−2.0087716
C	1.9183222	−3.7308445	−0.6327402
H	1.3955952	−4.4383484	0.0137720
H	1.4028486	−4.7634953	−2.4692790
H	3.7290481	−1.1807218	−2.9284307
H	4.7658850	1.4011674	−2.4687044
H	1.1804937	3.7227310	−2.9312571
H	−1.1792787	−3.7253970	−2.9302646
H	−4.7636375	−1.4018094	−2.4701453
H	−3.7320908	1.1831012	−2.9281398
H	−1.4044056	4.7648802	−2.4688076
N	3.1904527	2.5713907	−4.2814973
O	2.3867232	3.2056705	−4.9726749
O	4.1627278	1.9509215	−4.7318592
N	2.5775529	−3.1893476	−4.2803378
O	3.2130928	−2.3858263	−4.9705956
O	1.9573088	−4.1613317	−4.7316091

N	−3.1884944	−2.5743683	−4.2817031
O	−2.3850237	−3.2097826	−4.9721591
O	−4.1602788	−1.9537196	−4.7328211
N	−2.5815589	3.1924393	−4.2797546
O	−3.2189880	2.3902293	−4.9698331
O	−1.9609051	4.1641348	−4.7310760

CH_3CN@13

N	−2.0341957	1.0137312	1.6708704
H	−1.3757117	0.6795925	0.9705979
C	−3.1043997	0.2772563	2.1401974
C	−3.8557177	1.1226117	2.9391260
H	−4.7650406	0.8580981	3.4674120
C	−3.2288317	2.4019298	2.9263209
H	−3.5665919	3.2836747	3.4591232
C	−2.1056005	2.3196602	2.1179211
C	−1.0980189	3.3769357	1.7170504
N	1.0344266	2.0276131	1.6492005
H	0.7286434	1.4809214	0.8463769
C	0.2926864	3.0394800	2.2271797
C	1.1218374	3.6782120	3.1340999
H	0.8480506	4.5161555	3.7657342
C	2.3985543	3.0462927	3.0745053
H	3.2713020	3.3097534	3.6617847
C	2.3285232	2.0333909	2.1312354
C	3.3839692	1.0822022	1.6045791
N	2.0537251	−1.0676004	1.6038168
H	1.4045190	−0.7324370	0.8941294
C	3.1257868	−0.3368014	2.0773024
C	3.8717699	−1.1871891	2.8760515
H	4.7783560	−0.9268348	3.4110976
C	3.2380188	−2.4637340	2.8608590
H	3.5709508	−3.3489390	3.3911725
C	2.1161647	−2.3731870	2.0519771
C	1.1071756	−3.4235046	1.6351695
N	−1.0291410	−2.0728502	1.6062097
H	−0.7184155	−1.4936440	0.8281992
C	−0.2840268	−3.1004971	2.1524450
C	−1.1145924	−3.7758570	3.0309183
H	−0.8407623	−4.6361888	3.6316262
C	−2.3938006	−3.1490774	2.9883467

H	−3.2660983	−3.4365468	3.5646893
C	−2.3257068	−2.1018367	2.0829407
C	−3.3835026	−1.1209530	1.6200269
C	−1.5442489	4.7365357	2.2995980
H	−2.5351392	5.0028891	1.9119866
H	−0.8351170	5.5244328	2.0203670
H	−1.5940262	4.6865313	3.3950634
C	4.7742510	1.5435272	2.0968190
H	4.8195744	1.5287505	3.1938399
H	4.9746031	2.5641802	1.7485657
H	5.5531634	0.8770786	1.7062787
C	1.5535015	−4.7969589	2.1857048
H	2.5460096	−5.0523401	1.7947937
H	0.8444853	−5.5759976	1.8810563
H	1.5996554	−4.7749136	3.2823952
C	−4.7646488	−1.5926297	2.1290553
H	−4.9801538	−2.5982400	1.7476216
H	−5.5493861	−0.9090125	1.7831385
H	−4.7826388	−1.6179899	3.2265507
C	−0.9772725	3.5290373	0.1801476
C	0.0736033	4.3121971	−0.3334945
H	0.7487886	4.8153237	0.3615001
C	0.2954311	4.4169820	−1.7001483
C	−0.5230428	3.6916614	−2.5693035
C	−1.6006460	2.9457928	−2.0992237
C	−1.8354849	2.8931385	−0.7252434
H	−2.6804349	2.3161387	−0.3571780
C	3.4148986	1.0825750	0.0582852
C	3.9680645	−0.0175577	−0.6190259
H	4.3984784	−0.8332983	−0.0358363
C	3.9494039	−0.0945698	−2.0069809
C	3.3583501	0.9453788	−2.7266723
C	2.8633860	2.0815605	−2.0905624
C	2.9121088	2.1492675	−0.7002007
H	2.5193053	3.0314188	−0.1973795
C	1.0066423	−3.5482261	0.0963783
C	1.9740792	−3.0254062	−0.7712155
H	2.8635751	−2.5523793	−0.3606735
C	1.7944540	−3.0633483	−2.1531275
C	0.6448213	−3.6602306	−2.6672427
C	−0.2946366	−4.2699505	−1.8335931
C	−0.1077253	−4.2025378	−0.4587334

H	−0.8543377	−4.6343488	0.2108070
C	−3.4656208	−1.0362822	0.0768338
C	−2.9445735	−2.0276082	−0.7653992
H	−2.4923662	−2.9181489	−0.3338040
C	−2.9616502	−1.8764106	−2.1510043
C	−3.5511763	−0.7367229	−2.6953811
C	−4.1536252	0.2306663	−1.8891975
C	−4.0940704	0.0773584	−0.5092109
H	−4.5215913	0.8445242	0.1395365
C	0.0660062	−0.0933609	−1.7438134
N	0.0308794	−0.0440807	−0.5819912
C	0.1000529	−0.1595740	−3.1854857
H	0.1181144	0.8513241	−3.6193858
H	0.9867745	−0.7170205	−3.5188494
H	−0.7903647	−0.6854235	−3.5627888
H	−4.6346881	1.0916267	−2.3504313
H	−2.5227977	−2.6242083	−2.8109543
H	−1.1613378	−4.7623326	−2.2708401
H	2.5223338	−2.6254949	−2.8346793
H	4.3669893	−0.9467546	−2.5400913
H	2.4388574	2.8873136	−2.6868686
H	1.1073354	5.0192238	−2.1044220
H	−2.2358065	2.4099510	−2.8036465
N	0.3955846	−3.6206178	−4.1236462
O	−0.5499902	−4.2814957	−4.5622319
O	1.1399558	−2.9093749	−4.8087400
N	3.2273990	0.8264881	−4.1948265
O	3.7820405	−0.1308674	−4.7435116
O	2.5482127	1.6791244	−4.7765916
N	−0.2173554	3.6952362	−4.0149416
O	−0.7550576	2.8232855	−4.7096396
O	0.5641335	4.5510546	−4.4349012
N	−3.5208372	−0.5350365	−4.1577789
O	−2.7038575	−1.2051368	−4.8044662
O	−4.2939025	0.2944804	−4.6412610

Cl^{-}@**14**

Cl	−0.0002690	0.0002681	−0.2464608
N	2.3193457	−0.0342619	2.0560836
H	1.6414215	−0.0211610	1.2769718
C	2.9238396	1.0874244	2.5811173

C	3.8775616	0.6503185	3.4891070
H	4.5272852	1.2820131	4.0853569
C	3.8494368	−0.7741808	3.4917498
H	4.4749097	−1.4283950	4.0897189
C	2.8790317	−1.1778149	2.5862668
C	2.4775483	−2.5655077	2.1243486
N	−0.0437693	−2.3252268	2.0474944
H	−0.0275611	−1.6457652	1.2696394
C	1.0770331	−2.9181187	2.5891088
C	0.6372673	−3.8766358	3.4900732
H	1.2676082	−4.5217979	4.0927601
C	−0.7872004	−3.8625578	3.4733550
H	−1.4426686	−4.4929504	4.0646018
C	−1.1882464	−2.8955778	2.5624483
C	−2.5757927	−2.4901064	2.1052752
N	−2.3180618	0.0306853	2.0575554
H	−1.6406835	0.0190361	1.2779727
C	−2.9222525	−1.0919606	2.5808800
C	−3.8753215	−0.6565352	3.4903612
H	−4.5244842	−1.2893379	4.0860468
C	−3.8473034	0.7679677	3.4955041
H	−4.4724411	1.4210886	4.0950159
C	−2.8775209	1.1732519	2.5900943
C	−2.4763319	2.5618068	2.1305039
N	0.0449027	2.3214956	2.0518592
H	0.0280851	1.6434452	1.2727971
C	−1.0755529	2.9135488	2.5951157
C	−0.6352223	3.8706237	3.4973392
H	−1.2651719	4.5149552	4.1013211
C	0.7892385	3.8564106	3.4798343
H	1.4450806	4.4856700	4.0718731
C	1.1897137	2.8909984	2.5670239
C	2.5770014	2.4864122	2.1082615
C	3.4749973	−3.5952509	2.7019654
H	4.4900393	−3.3630607	2.3551354
H	3.2074054	−4.6035980	2.3614000
H	3.4647601	−3.5803199	3.8003717
C	−3.6029162	−3.4864709	2.6933045
H	−3.5766063	−3.4757108	3.7913429
H	−3.3764061	−4.5018179	2.3437385
H	−4.6140055	−3.2173631	2.3624951
C	−3.4734783	3.5904820	2.7105381

H	−4.4886983	3.3589289	2.3638046
H	−3.2060721	4.5994505	2.3716719
H	−3.4626764	3.5735399	3.8089108
C	3.6043844	3.4817930	2.6974851
H	3.3776353	4.4977393	2.3498200
H	4.6153176	3.2133257	2.3656757
H	3.5786577	3.4691122	3.7955174
C	2.5539925	−2.6690183	0.5859170
C	1.8012394	−3.6346999	−0.0959425
H	1.1635437	−4.3096771	0.4766947
C	1.8402115	−3.7305051	−1.4853493
C	2.6369273	−2.8413004	−2.2057961
C	3.4388826	−1.9128119	−1.5474896
C	3.3983480	−1.8370128	−0.1571312
H	4.0098206	−1.0981174	0.3587140
C	−2.7101491	−2.5805152	0.5684843
C	−3.7179873	−1.8657288	−0.0933501
H	−4.3612223	−1.2012940	0.4856217
C	−3.9246669	−2.0033482	−1.4651181
C	−3.0987498	−2.8579038	−2.1989059
C	−2.0989809	−3.5898627	−1.5594506
C	−1.9131683	−3.4501756	−0.1847488
H	−1.1337420	−4.0252577	0.3131818
C	−2.5536378	2.6680800	0.5922940
C	−3.3983883	1.8373645	−0.1517518
H	−4.0096122	1.0976249	0.3631645
C	−3.4395808	1.9154776	−1.5419512
C	−2.6379261	2.8450592	−2.1990875
C	−1.8408433	3.7330292	−1.4775304
C	−1.8012191	3.6349046	−0.0882965
H	−1.1633385	4.3090070	0.4851662
C	2.7103924	2.5796421	0.5715471
C	1.9126678	3.4503796	−0.1796517
H	1.1334714	4.0244428	0.3197885
C	2.0974706	3.5924129	−1.5542396
C	3.0969672	2.8617873	−2.1956479
C	3.9236505	2.0062335	−1.4638891
C	3.7179521	1.8662331	−0.0922033
H	4.3616340	1.2008243	0.4851601
H	4.7254993	1.4649012	−1.9663196
H	1.4843891	4.2789999	−2.1386173
H	−1.2459115	4.4762213	−2.0091918

H	−4.0719778	1.2469124	−2.1242634
H	4.0710504	−1.2433121	−2.1289774
H	1.2449870	−4.4727717	−2.0179763
H	−1.4864276	−4.2755665	−2.1454232
H	−4.7267287	−1.4609824	−1.9660859
O	2.6880134	−2.8967975	−3.6024594
O	3.3020982	3.1093584	−3.5529285
O	−2.6898163	2.9029788	−3.5956121
O	−3.3049210	−3.1030270	−3.5564716
C	−1.6530076	2.3796063	−4.3546979
O	−1.7602327	2.4268825	−5.5576324
C	−3.3103555	−2.1061617	−4.5213855
O	−3.6485567	−2.4155470	−5.6385281
C	1.6507928	−2.3720189	−4.3599819
O	1.7573156	−2.4170595	−5.5630721
C	3.3070812	2.1142175	−4.5196031
O	3.6439370	2.4257659	−5.6365541
C	−2.8793484	−0.7160880	−4.1213886
H	−3.7739039	−0.1021003	−3.9501644
H	−2.2669410	−0.6853251	−3.2139787
H	−2.3517753	−0.2729671	−4.9733254
C	0.4760985	−1.7870478	−3.6190703
H	−0.1617872	−2.5924076	−3.2284402
H	0.7591303	−1.1800182	−2.7477184
H	−0.1042337	−1.1980041	−4.3328637
C	2.8773043	0.7231707	−4.1216476
H	3.7724469	0.1095011	−3.9523462
H	2.2656653	0.6904189	−3.2137886
H	2.3492975	0.2812094	−4.9739222
C	−0.4778758	1.7932883	−3.6155417
H	0.1603162	2.5979694	−3.2240117
H	−0.7603443	1.1847817	−2.7450466
H	0.1019697	1.2054377	−4.3307195

CH_3CN@14

N	−1.4876919	1.6443928	2.4935057
H	−1.0569267	1.1667003	1.7046692
C	−2.7030208	1.2844349	3.0383112
C	−3.0412434	2.2751942	3.9461869
H	−3.9342576	2.2969519	4.5614353
C	−2.0093745	3.2604974	3.9197330

H	−1.9759556	4.1684383	4.5119442
C	−1.0575434	2.8558319	2.9966677
C	0.1919426	3.5423278	2.4773678
N	1.7561699	1.5540232	2.4545567
H	1.1867037	1.0337613	1.7895609
C	1.4528342	2.8185813	2.9149948
C	2.5418395	3.2424274	3.6610607
H	2.6295563	4.1928501	4.1761682
C	3.5256255	2.2099699	3.6208998
H	4.4981397	2.2331939	4.1003103
C	3.0218586	1.1724087	2.8513645
C	3.6641104	−0.1114613	2.3581596
N	1.6954232	−1.6942508	2.4257029
H	1.2105577	−1.2084715	1.6718750
C	2.9448858	−1.3392718	2.8895167
C	3.3468228	−2.3412567	3.7590116
H	4.2802788	−2.3695840	4.3107506
C	2.3168739	−3.3284225	3.7905773
H	2.3252346	−4.2434905	4.3727947
C	1.3031676	−2.9133820	2.9400407
C	0.0180655	−3.5934205	2.5036923
N	−1.5449708	−1.6082398	2.5513248
H	−1.0142471	−1.1028477	1.8432188
C	−1.2088629	−2.8613820	3.0190494
C	−2.2486187	−3.2734632	3.8383558
H	−2.3020672	−4.2146222	4.3748357
C	−3.2376162	−2.2450275	3.8364710
H	−4.1782812	−2.2604030	4.3761075
C	−2.7856081	−1.2223880	3.0159678
C	−3.4539786	0.0585727	2.5512317
C	0.2277512	4.9896803	3.0160747
H	−0.6710749	5.5295066	2.6925893
H	1.1105078	5.5128628	2.6277376
H	0.2698322	4.9967141	4.1136306
C	5.1358193	−0.1459659	2.8247007
H	5.1987367	−0.1178967	3.9210056
H	5.6766784	0.7174153	2.4174370
H	5.6201681	−1.0639337	2.4692124
C	0.0096825	−5.0395553	3.0457134
H	0.8820381	−5.5847152	2.6640002
H	−0.8997262	−5.5590676	2.7183496
H	0.0404574	−5.0452598	4.1437247

C	−4.8965131	0.0971859	3.1024037
H	−5.4613253	−0.7666774	2.7298919
H	−5.3999555	1.0151328	2.7739906
H	−4.8953146	0.0714719	4.2004596
C	0.1640599	3.6301672	0.9285740
C	1.3526499	3.8238399	0.2089530
H	2.2937457	3.8959700	0.7571213
C	1.3575903	3.9300610	−1.1836958
C	0.1464845	3.8318123	−1.8664197
C	−1.0527535	3.6634905	−1.1811190
C	−1.0378583	3.5774987	0.2106493
H	−1.9783604	3.4545775	0.7449777
C	3.6641213	−0.1557857	0.8071569
C	3.8631708	−1.3716389	0.1351455
H	4.0166539	−2.2798551	0.7209724
C	3.8603394	−1.4436334	−1.2590763
C	3.6450699	−0.2774512	−1.9905972
C	3.4724495	0.9475323	−1.3562452
C	3.4983812	1.0015992	0.0373178
H	3.3684030	1.9612652	0.5328591
C	−0.0679411	−3.6758917	0.9560949
C	1.0674928	−3.5713470	0.1440576
H	2.0437939	−3.4238015	0.6011127
C	0.9714289	−3.6319525	−1.2462355
C	−0.2714597	−3.8346096	−1.8332419
C	−1.4183236	−3.9857306	−1.0563112
C	−1.3054388	−3.8993189	0.3322904
H	−2.1971820	−4.0064159	0.9530067
C	−3.5483019	0.1012359	1.0032800
C	−3.4262753	−1.0555852	0.2243968
H	−3.2409565	−2.0120960	0.7079204
C	−3.5137107	−1.0045991	−1.1659951
C	−3.7593148	0.2140942	−1.7892440
C	−3.9199667	1.3832475	−1.0460775
C	−3.8061256	1.3137769	0.3446064
H	−3.9204443	2.2229253	0.9377412
H	−4.1285612	2.3269362	−1.5465134
H	−3.3670388	−1.8914116	−1.7797271
H	−2.3808838	−4.1674456	−1.5328185
H	1.8431704	−3.5025336	−1.8839008
H	−1.9775392	3.5795375	−1.7497219
H	2.2848774	4.0853501	−1.7321482

H	3.2963158	1.8364967	−1.9600990
H	4.0177558	−2.3898246	−1.7747707
O	0.0383872	3.9845183	−3.2601085
O	−3.9235199	0.1613556	−3.1862216
O	−0.2932767	−3.9910034	−3.2335503
O	3.6782284	−0.2554635	−3.3980697
N	0.0701516	−0.0735679	0.4915393
C	−0.0241880	0.0852290	−0.6555996
C	−0.1474843	0.3058264	−2.0640159
H	0.7868888	0.7355346	−2.4459313
H	−0.3645617	−0.6442927	−2.5696109
H	−0.9683457	1.0132650	−2.2454816
C	−3.1651945	0.9261435	−4.0215163
O	−2.4211358	1.8205303	−3.6643007
C	−0.9044660	−3.0588685	−4.0184456
O	−1.5801911	−2.1372179	−3.6020408
C	0.7071697	3.1593372	−4.1172984
O	1.5587942	2.3556821	−3.7874769
C	2.6794890	−0.8599540	−4.1111321
O	1.8329630	−1.5861024	−3.6301964
C	0.1909962	3.3641915	−5.5141975
H	−0.8035315	2.8945907	−5.5598647
H	0.0663223	4.4307074	−5.7376471
H	0.8628138	2.8913535	−6.2367611
C	2.7659659	−0.4390450	−5.5520145
H	2.4051256	0.5996983	−5.6028961
H	3.8034868	−0.4514072	−5.9076515
H	2.1345495	−1.0827619	−6.1718523
C	−0.5843140	−3.3213072	−5.4640612
H	0.4408652	−2.9613200	−5.6349084
H	−0.6081557	−4.3937522	−5.6907080
H	−1.2778081	−2.7713911	−6.1075464
C	−3.3475041	0.4564764	−5.4380825
H	−2.7705135	−0.4754328	−5.5396616
H	−4.3982748	0.2240053	−5.6487925
H	−2.9668380	1.2078852	−6.1365166
